# Volatilized Metabolites Produced by Soilborne *Aspergillus flavus* Regulate Fungal Conidiation, and Production of Secondary Metabolites

**DOI:** 10.1007/s10886-025-01657-4

**Published:** 2026-01-22

**Authors:** Lina Castano-Duque, Imtiaz Ahmad, Steven W. Lloyd, Matthew D. Lebar, Carol Carter-Wientjes, Nathaniel B. McCartney, Jared G. Ali, Geromy G. Moore

**Affiliations:** 1 USDA, Agriculture Research Service, Southern Regional Research Center, New Orleans, LA 70124 USA; 2 Center for Chemical Ecology, The Pennsylvania State University, State College, PA 16802 USA

**Keywords:** *Aspergillus*, Volatile organic compounds, Soil, Saprophyte, Terpenoids, Caryophyllene

## Abstract

Corn plants are susceptible to infection by fungi such as *Aspergillus flavus* that have the potential to produce several types of mycotoxins, one of the most potent ones known to exist is aflatoxin. Since aflatoxin contaminated corn must be destroyed, causing annual monetary losses in billions of dollars, it would be helpful to detect the presence of aflatoxin producing fungi at pre-harvest and pre-planting stages. To this end, it may be possible to capture volatile organic compounds these fungi produce while overwintering in the soil or while infecting corn plants. In this study, we acquired *A. flavus* VOCs from conidia and sclerotia, while residing in the soil environment. We concluded that soilborne conidia and sclerotia have specific VOC signatures, and sclerotia tend to emit specific VOCs at greater abundance compared to soilborne conidia or fungus-free soil. These sclerotium-specific VOCs include geranyl acetone, caryophyllene compounds, and methanone. Bioassays using toxin producing and non-producing fungi showed that β-caryophyllene increased spore concentration, and both β-caryophyllene and caryophyllene oxide were able to modulate aerial hyphae, sclerotia production and decrease aflatoxin, cyclopiazonic acid and ditryptophenaline production in *A. flavus* strains. Our results show insights into the ecological roles that β-caryophyllene and caryophyllene oxide have over *A. flavus’* development and metabolism, although their effect on multitrophic interactions among corn, fungi and other potential organisms are still not fully understood. These VOCs could serve as an agricultural surveillance management system that enables early mitigation of aflatoxin producing fungi outbreaks in the field.

**Supplementary Information:**

The online version contains supplementary material available at 10.1007/s10886-025-01657-4.

## Introduction

Fungi produce many metabolites that allow them to adapt to environmental changes, to communicate, to protect themselves and to survive (Yu et al. 2023; Keller 2019). Some of these metabolites are small molecules that go into the gas phase as volatile organic compounds (VOCs) (Hung et al. 2015). Certain VOCs are shared among different fungal genera (Mousa and Raizada 2013), yet many are unique to a genus, to a species, or to subset of strains within a species (Shankar and Sharma 2022; Moore and Lloyd 2023, 2025). Therefore, it is possible to capture, identify and utilize the unique VOCs as signatures (*i.e.*, biomarkers) to detect the presence of specific fungi in any given environment.

Fungi from the genus *Aspergillus* section *Flavi* are considered opportunistic pathogens whose preferred hosts include oil seed crops such as corn, peanut and various tree nuts (Liu et al. 2016; Rushing and Selim 2019). They can adversely affect plant hosts, particularly when the plants are stressed or weakened, often due to severe wounding or extreme drought conditions (Scheidegger and Payne 2003). *Aspergillus* species most often linked to infection of agricultural commodities include *A. flavus* and *A. parasiticus*, but there are 19 other species from section *Flavi* that have been isolated from various agricultural commodities (Frisvad et al. 2019). Several species within this genus produce sclerotia as overwintering structures to survive until conditions are favorable (i.e., the presence of a food source) for them to germinate, grow and sporulate (Wicklow 1987). Sclerotia also serve as reproductive structures, housing recombinant ascospores that result from sexual outcrossing between compatible strains (Horn et al. 2016). *Aspergillus flavus* strains may produce only large diameter sclerotia (≥ 400 µm; L-type), only small diameter sclerotia (< 400 µm; S-type), or they produce a mixture of L- and S-type sclerotia (M-type) (Cotty 1989; Sweany et al. 2024). The greatest impact from a fungus like *A. flavus* is not the rot it may cause on the plant, but the potential for subsequent contamination of crops with carcinogenic secondary metabolites known as aflatoxins. Eighteen of the 21 agricultural aspergilli in section *Flavi* produce aflatoxins (Frisvad et al. 2019), and there are at least 18 types of aflatoxins known to exist. Within sclerotia of these fungi, aflatoxins are produced at higher concentrations by S-type aspergilli than in L-type (Cotty 1989). The most serious being aflatoxin B_1_, which is considered the most potent natural compound known to exist (Abrehame et al. 2023). This mycotoxin has been linked to deaths of animals (Zijden et al. 1962) and humans (Probst et al. 2007), as well as global economic losses in the billions of dollars annually (Shabeer et al. 2022). To reduce risks to consumer health, certain countries, such as the U.S.A. and the E.U., enforce strict regulations on the permissible levels of aflatoxin in food and animal feed. However, these regulations do not prevent the economic losses growers may incur during an aflatoxin outbreak, as contaminated crops must be discarded.

Since the Turkey X outbreak in the 1960 s exposed the devastating impact of aflatoxin contamination on livestock, protecting key agricultural commodities has been a major focus of scientific research (Blount 1961). This event catalyzed advancements in crop protection, leading to strategies such as chemical fungicide applications and genetic breeding for resistance against aflatoxigenic fungi (Rajasekaran et al. 2006; Masiello et al. 2019). The most popular strategy to date is the pre-harvest application of naturally non-aflatoxigenic *A. flavus* as biocontrol agents to fields where aflatoxin contamination is possible. This is an effective strategy that is being adopted globally. Commercially available formulations now exist in high income countries like the U.S.A. and E.U., as well as low to middle income countries like those in Africa (Regnault-Roger 2012; Atehnkeng et al. 2014; Mauro et al. 2018; Ortega-Beltran et al. 2019; Savić et al. 2020). However, there is still a need for additional pre- and post-harvest strategies to enhance food/feed security. Additionally, the exact mechanism of control has yet to be fully elucidated. A new area of biocontrol research has found the potential for chemosensing to be one mechanism of biocontrol, whereby non-aflatoxigenic *A. flavus* strains produce VOCs that can inhibit growth and/or toxin production (Moore et al. 2021, 2022). Another way to help growers would be to exploit VOCs to facilitate detection of aflatoxigenic *A. flavus* as early as possible, either in the soil or early in the crop infection process. This would allow growers to treat the soil ahead of planting, or to target the infected plant(s) in the field for removal/destruction before harvest of the entire field, which could expose healthy seeds to *A. flavus* infected (and aflatoxin contaminated) seeds.

Capturing and identifying VOCs produced by aflatoxigenic *Aspergillus flavus*—both during overwintering in soil and throughout host crop infection—represents a critical first step in developing early detection strategies. By building a comprehensive library of these VOCs, we can establish reliable biomarkers that may later inform sensor development and machine learning (ML) models for improved fungal monitoring. This foundational work lays the groundwork for future technologies that could enhance pre-planting and pre-harvest assessments, ultimately reduce grower losses and improve food safety. Moore and Lloyd (2023 and 2025) conducted *in vitro* studies in which they captured and identified VOCs from different aflatoxigenic fungi while growing on several types of synthetic media, as well as kernels harvested from susceptible and resistant corn genotypes, to build a biomarker library (Moore and Lloyd 2023, 2025). To build on these findings, in this study we conducted growth chamber experiments using two different methods to capture VOCs emanating from soil inoculated with either *A. flavus* conidia or *A. flavus* sclerotia. Furthermore, we identified compounds that are significantly higher in sclerotia and performed bioassays to determine the potential biological and ecological role of those VOCs.

## Methods and Materials

### Experimental Set-up

To prepare the fungal inoculum, multiple PDA (Potato Dextrose Agar) plates were inoculated with *A. flavus* strain NRRL 3357 (Nierman et al. 2015). The cultures were grown in darkness at 31 °C for 7 d, before harvesting conidia and sclerotia. The conidia were harvested to make a spore suspension with a final concentration of 1 × 10^4^ CFU/ml. To harvest the sclerotia, all remaining conidia were rinsed away with 0.01% Triton –X-100. The sclerotia were gently dislodged from the agar surface and transferred to a 15 ml tube containing sterile water and vortexed to dislodge the residual (and hydrophobic) conidia. The water was drawn off and discarded, fresh water was added, and the process was repeated two more times. The sclerotia were then transferred to petri dishes containing filter paper and allowed to dry. The spore suspension and sclerotia were held at 4 °C until needed. We acquired and cleaned ten 47 mm glass petri dishes (Corning Inc., Glendale, AZ, U.S.) and glass domes (130 mm OD x 200 mm Height, 6.5–8.5 mm OD hose connection on top and near the bottom at 90°; Chemglass Life Sciences, NJ, U.S.). Metro-Mix professional growing mix soil was acquired from Sun Gro Horticulture (Agawam, MA, U.S.), autoclaved and allowed to cool before placing 2.5 g into each of the glass petri dishes. We then added 4 ml of water and measured relative humidity (RH) with a soil moisture meter (Spectrum Technologies Inc., Aurora, IL, U.S.), which was at an approximate level of “6” which is described as “average wet” by the equipment manufacturer. Two types of VOC collection were conducted. One collection method involved Solid Phase Microextraction (SPME) fibers (SPME Arrow with DVB/Carbon wide range/PDMS coating, (SPME Arrow with DVB/Carbon wide range/PDMS coating, Restek Corp., Bellefonte, PA, U.S.). In preparation for their use, the SPME fibers were conditioned for 5 min by exposure to flowing nitrogen at 270 °C. The second collection method involved filter traps containing HayeSep Q adsorbent polymers (Hayes Separation Inc., Bandera, TX, U.S.).

### Soil VOC Collections

The VOC collection experiments included five replicates of soil inoculated either by pipetting a 0.2 ml spore suspension of conidia, or placing approximately 20–30 sclerotia, onto the soil surface and then covering them with a lid. The control replicates for the conidium study received only 0.2 ml of autoclaved distilled water, while the five control plates for the sclerotium study were soil only. For both sets of experiments, the petri dishes were incubated at 30 °C in darkness for 7 d. On day 7, the petri dishes were uncovered and placed under a glass dome to allow VOCs to fill the headspace. SPME fibers were placed inside the dome through a side spigot and exposed to available VOCs for 1 h. After SPME collection, a pull/push air system was set up using the same glass domes but with the HayeSep Q traps, which collected available VOCs for approximately 6 h (Supplemental Fig. 1). Additional VOC collections using empty glass domes (no soil, conidia or sclerotia) were also conducted to serve as blanks.

### VOC Qualification and Quantification from SPME Fibers

Exposed SPME SPME fibers were desorbed using the injection port of an Agilent 8890 GC equipped with a PAL RSI 85 autosampler and a 5977B mass selective detector (MSD) (Agilent Technologies, Santa Clara, CA, U.S.). The fiber was desorbed for 2 min at 270 °C. The helium carrier gas pressure was set to 50 psi for 2 min in splitless mode. After desorption, the fiber was withdrawn, and the column flow was set at 1 ml/min. The column was an HP-5MS UI, 25 m long, 0.25 mm internal diameter (Agilent Technologies, Santa Clara, CA, U.S.). The fiber was then conditioned for 5 min in flowing nitrogen at 270 °C. The oven was held at 35 °C for 2 min, ramped at 5 °C/min to 100 °C, then at 10 °C/min to 200 °C, then at 25 °C/min to 270 °C, and the held at 270 °C for 3 min. The total run time was 30.8 min. The column was attached to the MSD through a transfer line held at 280 °C. The source was held at 230 °C and the quadrupole at 150 °C. After a 1.4 min delay, data were collected using a 50 m/z threshold and a scan range of 35 to 350 m/z at 4.5 scans per sec.

### VOC Qualification and Quantification from HayeSep Q Filters

HayeSep Q traps were conditioned by first eluting by using 150 µl of methylene chloride and receiving 200 ng of the internal standard, nonyl acetate, prior to analysis using an Agilent 6890 GC coupled to an Agilent 5977B MSD (Agilent Technologies, Santa Clara, CA, U.S.). Splitless injections of 1 µl were made with the inlet at 230 °C and separations were performed on a HP-5MS UI column (30 m × 0.25 mm × 0.25 µm) using helium carrier gas at constant flow of 0.7 ml/min. The oven was held at 40 °C for 2 min and then increased by 10 °C/min to 300 °C. The MSD was operated in EI mode using standard parameters with temperatures and scan settings the same as those described above.

### VOC Analysis

MSD from both collection types were analyzed with MassHunter software (Agilent Technologies, Santa Clara, CA, U.S.) using the Unknowns Analysis package for peak deconvolution, component discovery, and creation of a custom mass spectral library documenting all observed analytes. A quantification method was generated from the custom mass spectral library using the MassHunter Quantitative Analysis package and then manually optimized to ensure that quantitative and qualitative ions were sufficiently distinguishable. All target peaks detected by MassHunter were manually reviewed to confirm accurate identity and integration. Resulting peak areas of analytes and the internal standard were used to calculate total emission for each analyte. Identifications were made using a combination of mass spectral library searches (NIST 17 and Adams), retention index matching, and, whenever possible, comparison to authentic standards. Analytes for which a definitive identification could not be made were named by their observed retention index using the prefix ‘RI’.

### Statistical Analysis of VOC Content

**Fig. 1:**
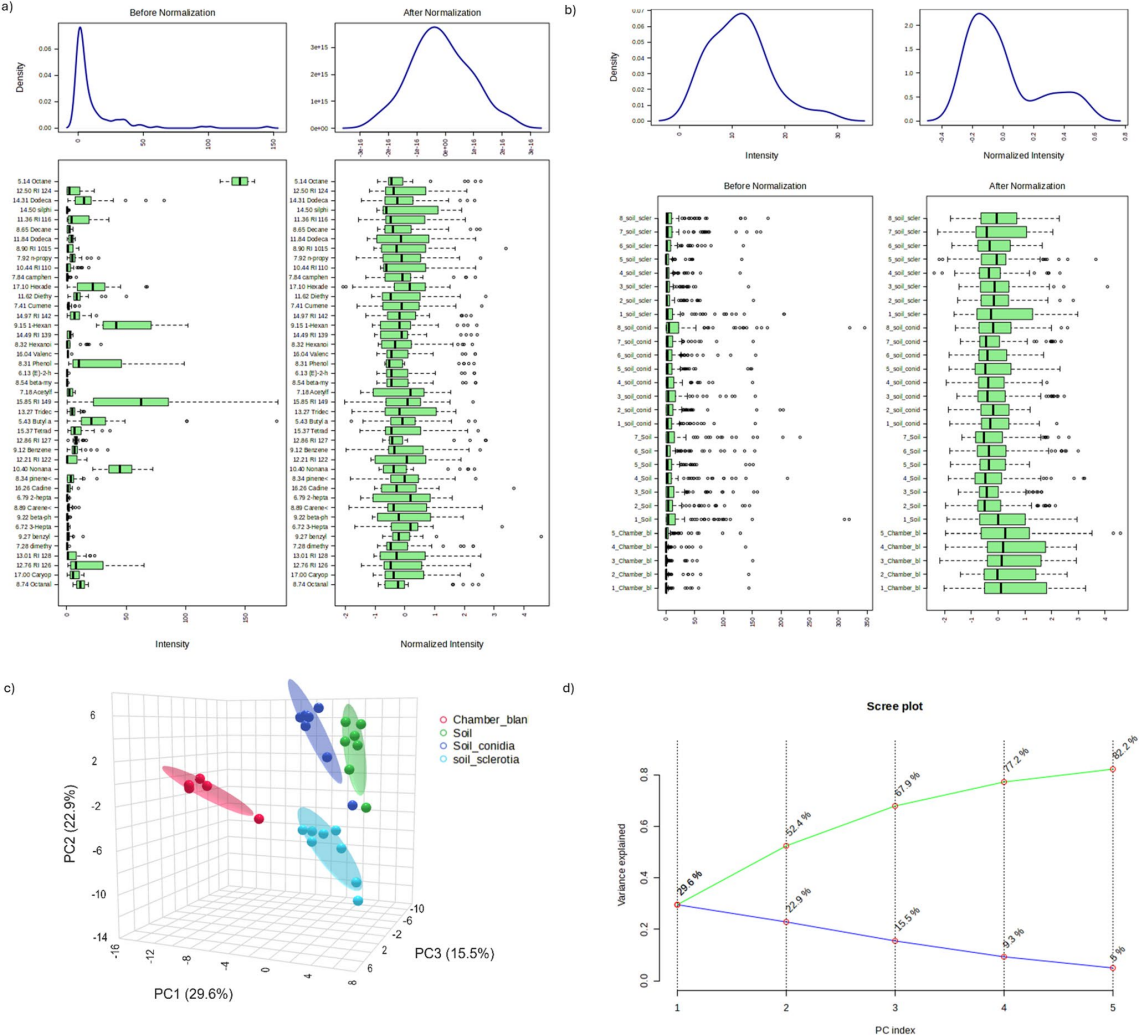
VOC data preparation and spatial variation analysis from HayeSep Q collection method. Before and after normalization of **a**) VOCs and **b**) samples. **c**) Principal component analysis and **d**) PC component loadings of variance. In PCA, red: Chamber blank, green: soil, dark blue: conidia, and light blue: sclerotia. In PC loadings, green: PC cumulative variance and blue: PC. variance

**Fig. 2:**
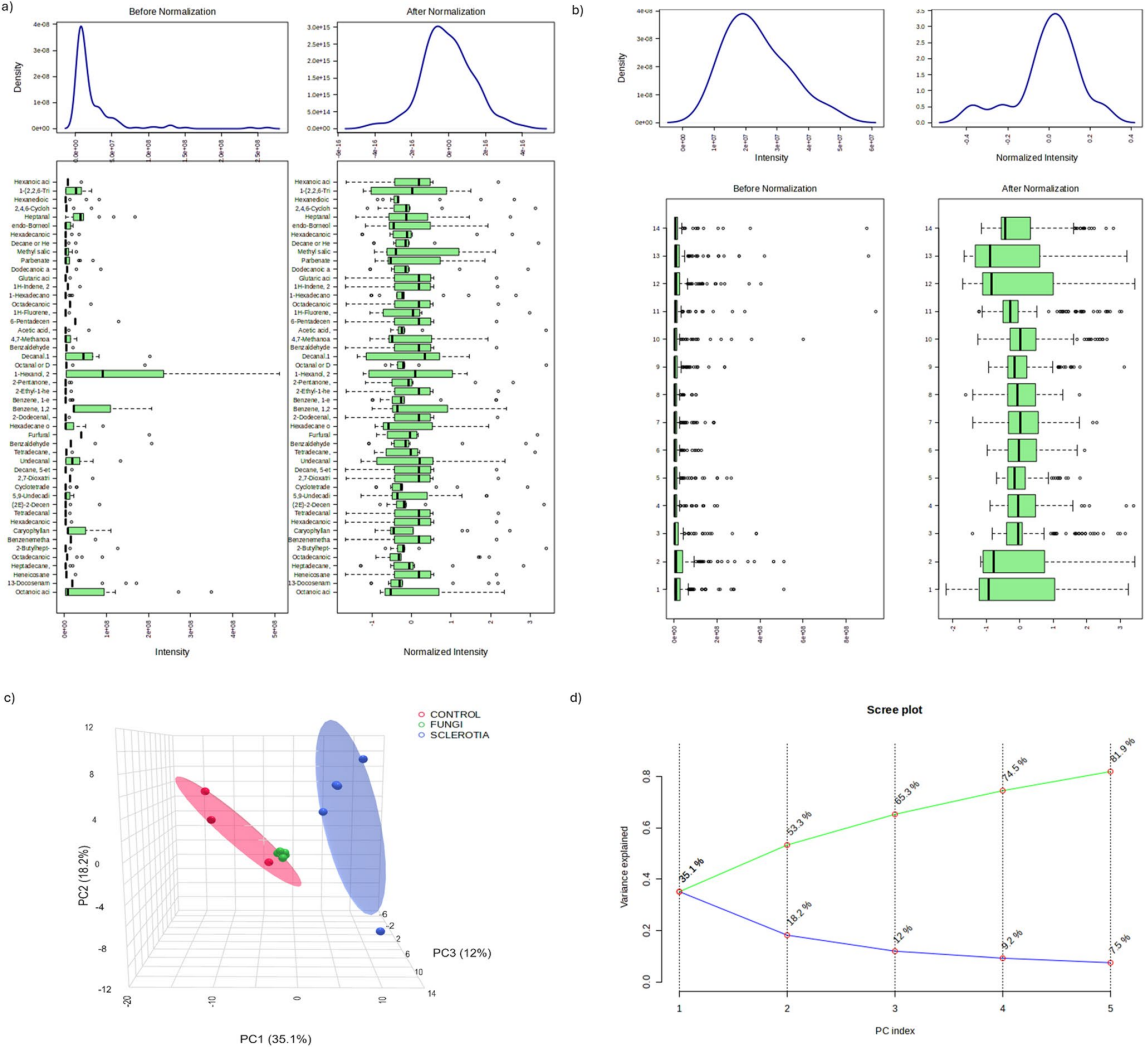
VOC data preparation and spatial variation analysis from SPME collection method. Before and after normalization of **a**) VOC and **b**) samples. **c**) Principal component analysis and **d**) PC component loadings of cumulative variance. In PCA, red: soil (Control), green: conidia, dark blue: sclerotia. In PC loadings, green: PC cumulative variance and blue: PC. variance

Area under the peak from the SPME and HayeSep Q collections were uploaded to MetaboAnalyst online software (www.metaboanalyst.ca/) and each VOC collection method was analyzed separately. Data pre-processing for soil samples (control, conidia, and sclerotia) was performed using a variance filter of interquartile range at 5%, that would filter out variables that were nearly constant throughout the experiment (Figs. 1a, b and 2a, b). This removed 13 compounds from the HayeSep Q dataset and eight compounds from the SPME dataset. Data were normalized using the median platform to reduce systematic differences among samples, subjected to square root transformation, and scaled with auto-scaling (mean-centered and divided by the standard deviation of each variable) (Figs. 1a, b and 2a, b). Using MetaboAnalyst, hierarchical clustering analysis was performed on the normalized data by using Pearson correlation method, followed by ANOVA and Tukey’s HSD-test. Pattern search analysis used specific VOC targets to find other VOCs that followed a linear relationship in abundance with the target VOC.

### Bioassays with VOCs

Following the protocols of VOC exposure studies conducted by Moore et al. (2021 and 2022) involving *A. flavus* (Moore et al. 2021, 2022), two strains were used for exposure to specific VOCs that were commercially available. One was aflatoxigenic strain NRRL 3357 (Nierman et al. 2015), herein called AF3357, and the other is a non-aflatoxigenic strain, AF36, which is a commercial biocontrol agent (Doster et al. 2014). In order to determine the biological roles of these VOCs, we exposed AF3357 and biocontrol AF36 to different concentrations of these compounds. Treatments with each compound required different concentrations because of their maximum solubility in DMSO. Therefore, the concentrations of β-caryophyllene used were 0.5 M, 1 M and 3.5 M, while those of caryophyllene oxide were 0.05 M, 0.1 M and 0.2 M. To prepare the fungal inoculum, both strains were incubated on PDA for 7 d and then spore suspensions of each were generated with final concentrations of 1 × 10^4^ CFU/ml. There were 5 replicate plates per experiment. Ten microliters of each strain were then single point inoculated onto the center of yeast extract sucrose (YES) agar plates. Two controls were used: no external compound present and DMSO (100%) since it was used to dissolve the pure VOC compounds. Experimental plates included a well to which 20 µl of the VOC solution of interest was pipetted. Three different concentrations of each of the VOC compounds were used to assess dose response, and concentrations were selected based on the maximum solubility of the compound in DMSO at room temperature. All plates were incubated in darkness for 7 d at 31 °C. Fungal growth was measured daily as colony diameter, and on day 7, the cultures were subjected to metabolite extraction for assessment of aflatoxin production.

### Spore Concentration, and Spore Size Measurements

After the five agar plugs were removed from the colonies, 20 ml of 0.1% Triton in distilled water were used to flood the plates and to scrape the fungal biomass. A dilution of 1:100 was used to measure spore concentration and spore size by using an Olympus R1 Cell Counter system (Cambridge Scientific, Watertown, MA, U.S.). Five replicate readings were taken to ensure consistency in enumeration.

### Aflatoxin Extraction and Measurements via UPLC Analysis

From each AF3357 and AF36 colony, five agar plugs (6 mm) were excised and placed in 4 ml glass vials for metabolite extraction with acetonitrile:water:formic acid (80:19:1, v/v/v, 1 ml). The contents were incubated on an orbital shaker (200 rpm) for 2 h at room temperature. The extracts were then centrifuged to pellet particulates, and the particulate-free extracts were transferred to clean tubes and analyzed (1 μl injections) using a Waters ACQUITY UPLC system (40% methanol in water, BEH C18 1.7 μm, 2.1 mm × 50 mm column) with fluorescence detection (Ex = 365 nm, Em = 440 nm). Some samples needed dilution to avoid saturating the detector. Identification and quantification utilized an analytical standard of aflatoxin B_1_ (AFB_1_) purchased from Sigma-Aldrich (St. Louis, MO, U.S.). AFB_1_ content was expressed in parts per billion or PPB (ng/g agar).

### A. flavus Secondary Metabolite Analysis

*A. flavus* secondary metabolites in the extracts were analyzed on a Waters Acquity UPLC system coupled to a Waters Xevo G2 XS QTOF mass spectrometer. Extract injections (1 µl) were separated on a Waters BEH C18 1.7 µm, 2.1 × 50 mm column with the following gradient solvent system: (0.5 ml/min, solvent A: 0.1% formic acid in water; solvent B: 0.1% formic acid in acetonitrile): 5% B (0–1.25 min.), gradient to 25% B (1.25–1.5 min.), gradient to 100% B (1.5–5.0 min.), 100% B (5.0–7.5 min.), then column equilibration to 5% B (7.6–10.1 min.). The Z-spray ionization source was run in ESI + mode using MassLynx 4.2 software with the following settings: source temperature: 100 °C, desolvation temperature: 250 °C, desolvation gas flow: 600 L/h, cone gas flow: 50 L/h, capillary voltage: 3.0 kV, sampling cone voltage: 40 V. Analyses were performed in sensitivity and continuum mode, with a mass range of m/z 50–1200 and a scan time of 0.1 s. A data-independent acquisition method with elevated collision energy (MSE) was used with 6 eV low energy and a high energy ramp from 15 − 45 eV. Mass data were collected from 2.0–6.0 min. then imported, analyzed, and quantified on Waters UNIFI 1.9.4 software using “Quantify Assay Tof 2D” analysis method with lock mass corrected by UNIFI. CPA standard was purchased from Sigma-Aldrich (St. Louis, MO, United States). Indole diterpene and other metabolite standards were purified from *A. flavus* cultures and sclerotia. Metabolite content is expressed in PPB (ng/g agar).

### Data Analysis of Fungal Growth, AFB_1_ Production and Spore Production

Fungal growth was calculated by fitting a Baranyi growth model (Baranyi and Roberts 1995) to the data for each concentration treatment using the *growthrates* package (Hall et al. 2014) in R (Team RC 2014). The Baranyi model considers that there is a lag phase for growth and is based on two differential equations (Baranyi and Roberts 1995). Using this model fitting, we were able to determine the growth parameters of initial growth (y_0_), growth rate (mu_max_) and maximum growth (K) for each strain tested. Differences in growth rate, spore concentration, spore size, fungal biomass, sclerotia weight/counts and AFB_1_ content among fungal strains and treatments were ascertained by performing One-Way ANOVA and the HDS-Tukey pairwise test.

## Results

Principal component analysis (PCA) showed that VOCs from control soil and soil inoculated with conidia tend to cluster together in the 3D-space (Figs. 1c, d and 2c, d), meaning that these two treatments have similar VOC profiles, while soil inoculated with sclerotia produced VOCs that were different and more specific to this stage of the *A. flavus* life cycle (*i.e*., overwintering). These results were observed with both the HayeSep Q and SPME collection methods (Figs. 1c, d and 2c, d). Cumulative variance visualization showed that the third PC-loading (PC3) explained 67.9% (Fig. 1d) and 65.3% (Fig. 2d) for HayeSep Q and SPME, respectively. In addition to PCA, we performed a Sparse partial least square discriminant analysis (SPLS-PCA) to reduce the number of VOCs in the data. This analysis confirmed the specificity of sclerotium-related VOCs compared to VOCs from control soil and soil containing conidia, indicating the top VOCs in PC1 from HayeSep Q and SPME collection methods (Supplemental Figs. 2 and 3). Among the VOCs in loading 1 of the PCA, those with the highest abundance in sclerotia were octadecane captured via the HayeSep Q collection method (Supplemental Fig. 2), as well as methanone, β-patchoulene, and 1-ethyl-4-methyl-benzene captured via the SPME collection method (Supplemental Fig. 3).

**Fig. 3:**
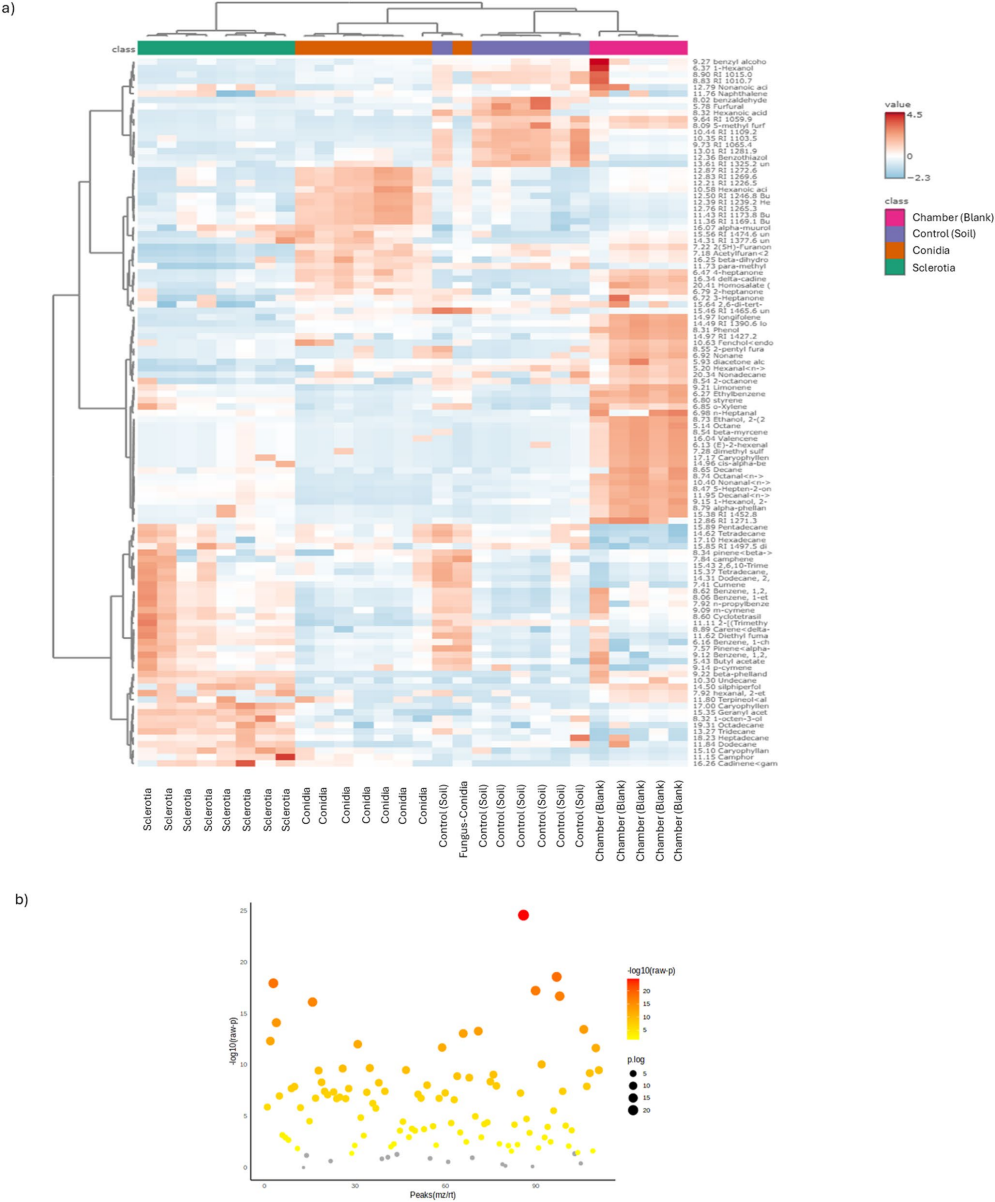
VOC quantitative analysis from HayeSepQ collection method. **a**) hierarchical clustering analysis and heatmap. **b**) ANOVA results. In clustering analysis, pink: chamber blank, purple: soil (Control), brown: conidia and green: sclerotia. In heatmap, ramp color represents abundance values after normalization, blue (low) to red (High). In ANOVA, the y-axis represents – logarithm base 10 of raw p-values and the x-axis represents peaks of metabolites (mz/rt). Diameter of the points represent the p-value logarithmic result and color ramp from yellow to red represent -logarithm base 10 of raw p-value

**Fig. 4:**
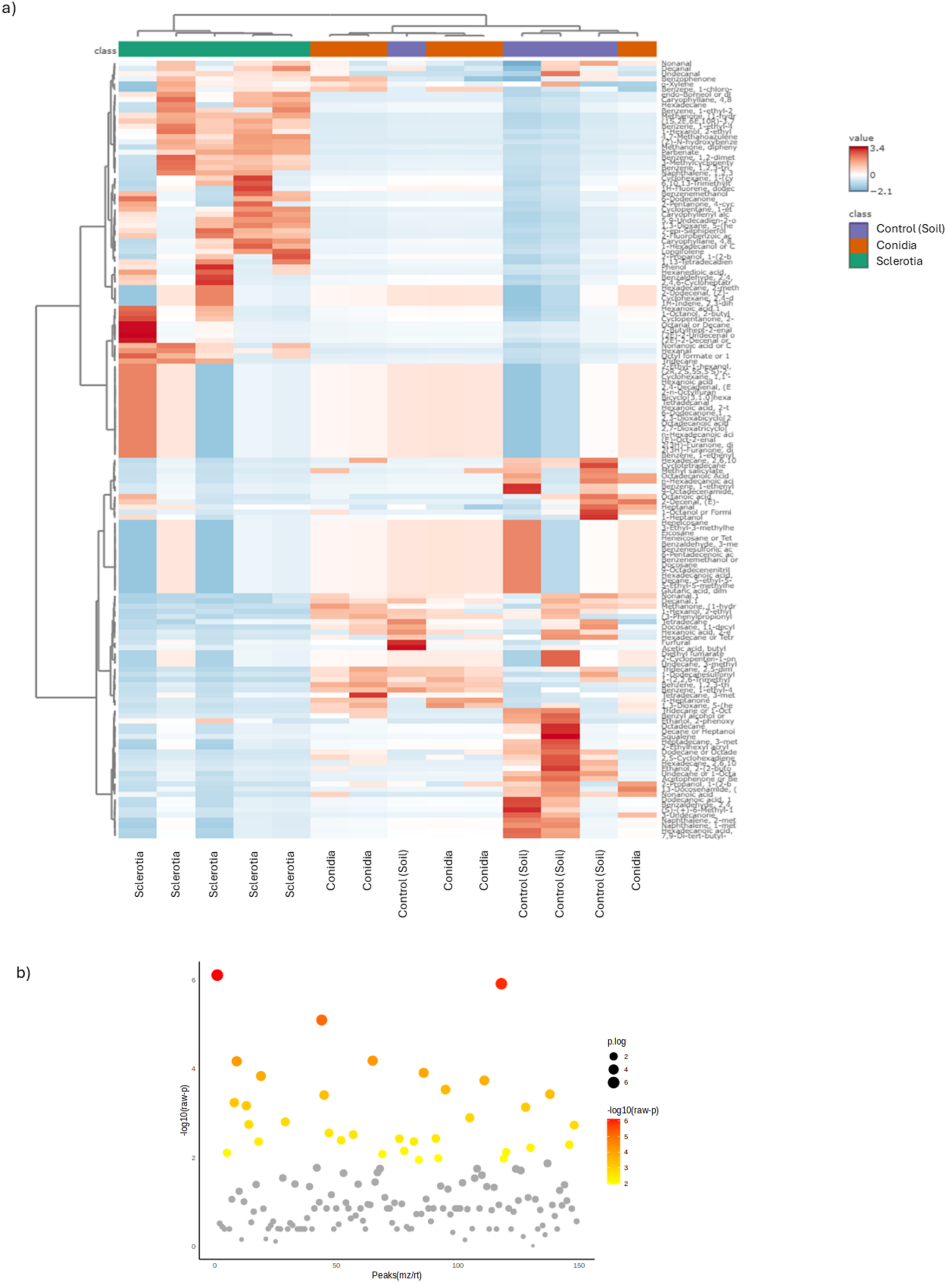
VOC quantitative analysis from SPME collection method. **a**) hierarchical clustering analysis and heatmap. **b**) ANOVA results. In clustering analysis, purple: soil (Control), brown: conidia and green: sclerotia. In heatmap, ramp color represents abundance values after normalization, blue (low) to red (High). In ANOVA, the y-axis represents – logarithm base 10 of raw p-values and the x-axis represents peaks of metabolites (mz/rt). Diameter of the points represent the p-value logarithmic result and color ramp from yellow to red represent -logarithm base 10 of raw p-value

Hierarchical clustering analysis showed similar results to the PCA and SPLS-PCA, whereby VOCs from soil-inhabiting sclerotia tend to cluster further from soil containing conidia or control soil (Figs. 3a and 4a). Clustering analysis showed that soil containing sclerotia tended to emit VOCs that were greater in abundance compared to soil containing conidia and no fungi. The greatest among those compounds was caryophyllene (Figs. 3a and 4a). Tukey’s HSD-test showed 97 VOCs with significant differences in abundance from HayeSep Q collections. Among these VOCs, some were more abundant in sclerotium treated soil compared to the other treatments, including tridecane and γ-cadinene (Supplemental Table 1). Tukey’s HSD-test showed 34 VOCs from SPME collections with significantly different abundances (Figs. 3b, 4b and Supplemental Table 1). Among these VOCs, several different VOCs were more abundant in sclerotium treated soil, such as decanal, hexanal, parbenate, and methanone (Supplemental Table 1). The most significantly abundant VOC in sclerotium samples, detected with both HayeSep Q and SPME methods, was caryophyllene (Supplemental Table 1).

**Fig. 5:**
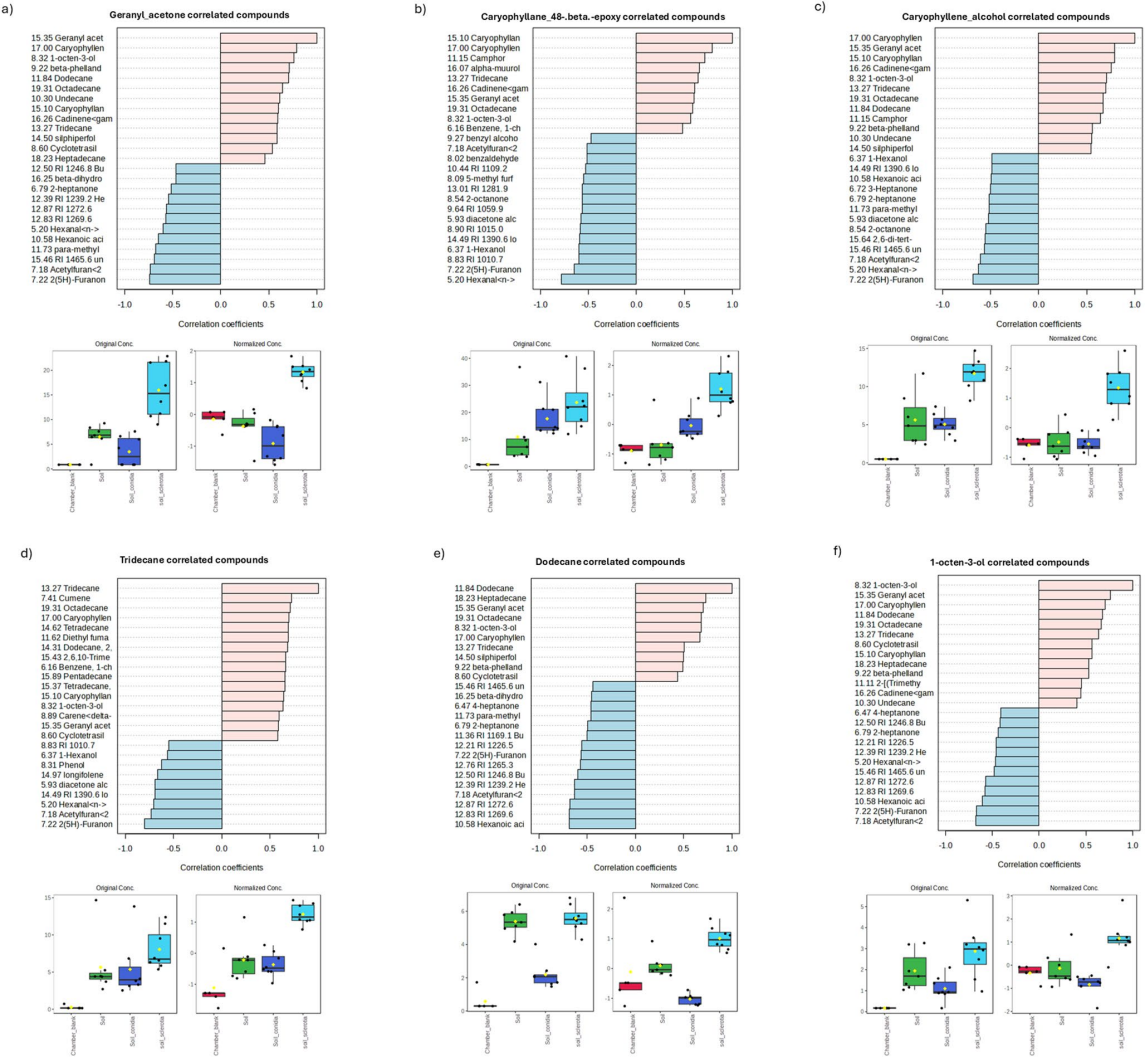
VOCs feature correlation analysis from HayeSep Q collection method. **a**) Geranyl acetone, **b**) Caryophyllane, 4,8-.beta.-epoxy, **c**) Caryophyllene alcohol, **d**) Tridecane, **e**) Dodecane and **f**) 1-octen-3-ol. Color in the feature correlation bars represent in pink a positive correlation and in blue a negative one. Box-plots represent the VOC abundance per treatment, left panel is raw-data and right panel is normalized-data, color of the boxes represent treatment, red: Chamber blank, green: soil, dark blue: conidia, and light blue: sclerotia. Box–Whisker plot depicts the maximum (25th – 1.5 * interquartile range “IQR”) and minimum [75th percentile + 1.5 *interquartile range (IQR)], and the Box–Whisker plot depicts median, first (25th percentile) and third (75th percentile) quantiles distribution

**Fig. 6:**
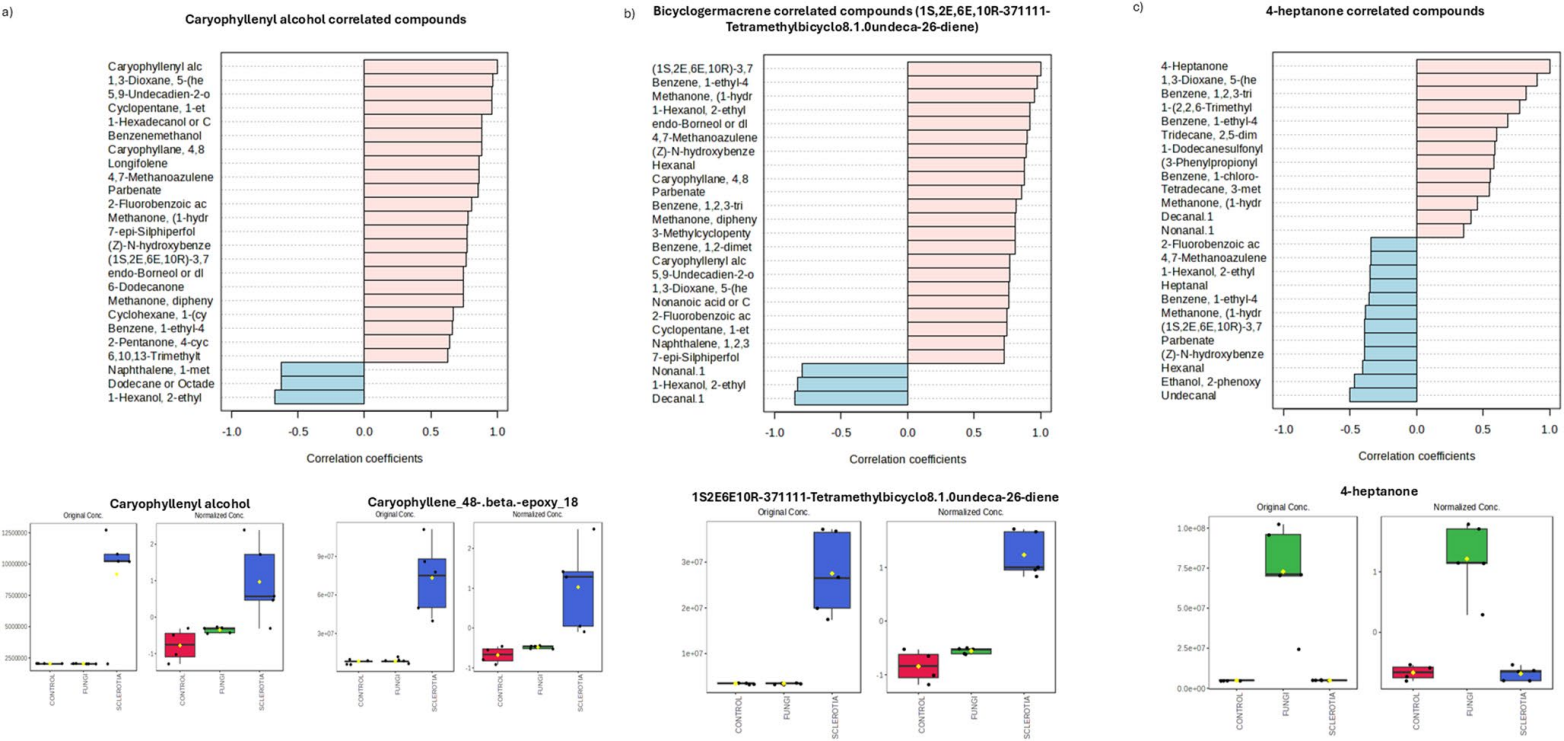
VOCs feature correlation analysis from the SPME collection method. **a**) Caryophyllene alcohol, **b**) bicyclogermacrene, **c**) 4-heptanone. Color in the feature correlation bars represent in pink a positive correlation and in blue a negative one. Box-plots represent the VOC abundance per treatment, left panel is raw-data and right panel is normalized-data, color of the boxes represent treatment, red: soil (Control), green: conidia, and dark blue: sclerotia. Box–Whisker plot depicts the maximum (25th – 1.5 * interquartile range “IQR”) and minimum [75th percentile + 1.5 *interquartile range (IQR)], and the Box–Whisker plot depicts median, first (25th percentile) and third (75th percentile) quantiles distribution

We performed a pattern detection analysis to determine if there were compounds with a similar abundance profile as caryophyllene. The HayeSep Q method detected compounds such as geranyl acetone, 4,8-beta-epoxycaryophyllene, and octadecane (Fig. 5, Supplemental Fig. 2, while SPME detected compounds such as methanone and 1-ethyl-1-methylcyclopentane (Fig. 6, Supplemental Fig. 3). We performed pattern detection on other compounds with high abundance in sclerotium treated soil, such as tridecane, dodecane and 1-octen-3-ol (Fig. 5), as well as bicyclogermacrene and 4-heptanone (Fig. 6), and found all of these compounds to have abundance profiles similar to caryophyllenyl alcohol. Like Tukey’s HSD analysis for HayeSep Q-detected VOCs, we observed the same VOCs following the high abundance pattern in sclerotia treated soil: tridecane and γ-cadinene (Supplemental Fig. 4). There were some unidentified VOCs with high abundance on soil control samples (RI_1109.2, RI_1281.9, 1103.5), as well as benzothiazole (Supplemental Fig. 4). Additionally, there were a group of VOCs with high abundance patterns associated only with conidia treated soil, including hexanoic acid and butanoic acid (Supplemental Fig. 4).

**Fig. 7:**
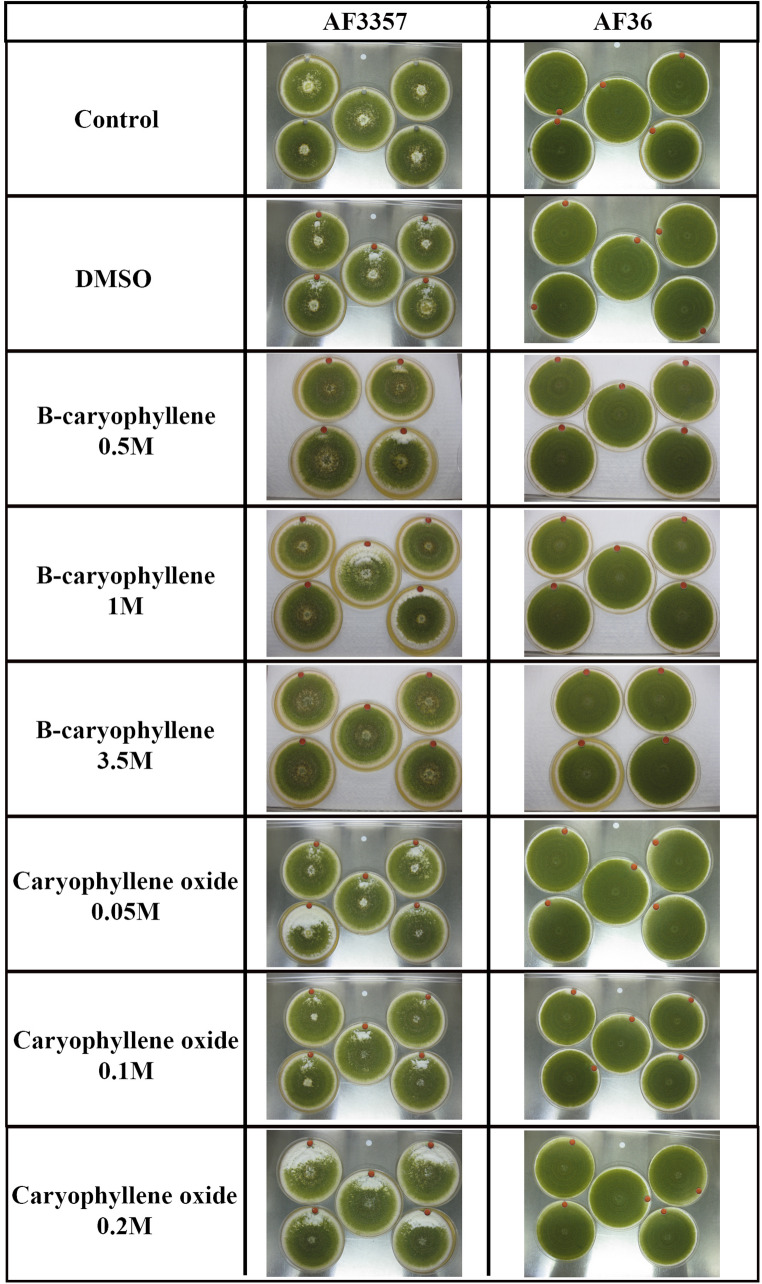
Visualization of fungi (AF3357 and AF36) treated with β-caryophyllene (0.5, 1 and 3.5M) and caryophyllene oxide (0.05, 0.1 and 0.2M). Controls were DMSO (100%) and empty liquid holder (Control)

**Fig. 8:**
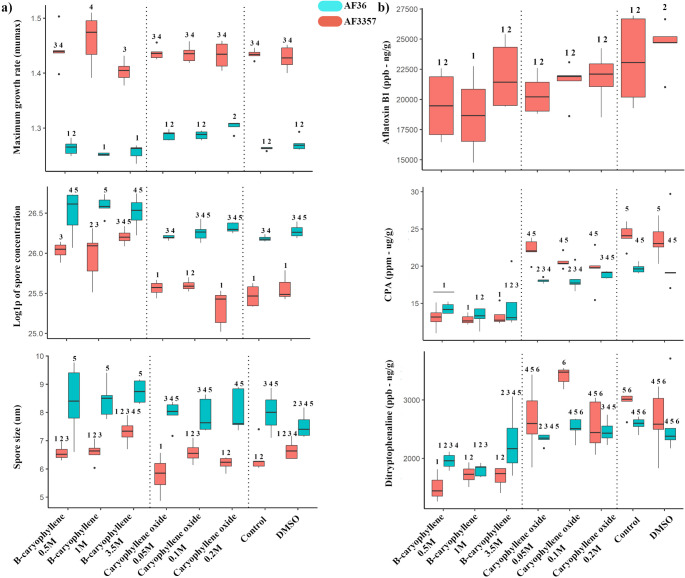
Phenotypic characteristics of fungi treated with VOCs. **a**) Maximum growth rate, spore density, size and **b**) mycotoxin concentration of fungi treated with β-caryophyllene (0.5, 1 and 3.5M) and caryophyllene oxide (0.05, 0.1 and 0.2M). Controls were DMSO (100%) and empty liquid holder (Control). Color of the boxes represents fungal strain, salmon: AF3357, blue: AF36. Box-plot whisker plot depicts the maximum (25th – 1.5 * interquartile range “IQR”) and minimum [75th percentile + 1.5 *interquartile range (IQR)], and the Box–Whisker plot depicts median, first (25th percentile) and third (75th percentile) quantiles distribution

For the bioassays, we selected two VOCs that exhibited significant differences in accumulation profiles: β-caryophyllene and caryophyllene oxide. These were used for several reasons: simplification of experiments (versus testing many compounds), relative significance, abundance in headspace, availability/affordability for purchase, and previous associations with a living organism (primarily plants or fungi) according to the literature. Visual analysis of the fungal colonies showed that AF36 was not noticeably affected by any of the treatments we used (Fig. 7). AF3357 inherently produces aerial hyphae in older parts of the colony, as evidenced at the center of each control plate. However, every treatment (including the DMSO control) caused AF3357 to produce additional aerial hyphae, mostly near the VOC source. Caryophyllene oxide noticeably enhanced production of aerial mycelia, compared to the other treatments (Fig. 7). Among the β-caryophyllene treatments involving AF3357, fewer aerial hyphae were produced with the 3.5 M concentration, even fewer than the AF3357 control colonies (Fig. 7). Overall, the maximum growth rate was significantly higher for AF3357 compared to AF36; however, there were no observable differences among treatments and controls (Fig. 8a).

Spore concentration analysis showed that AF36 and AF3357 control colonies were significantly different, with AF36 having greater spore concentrations. The only differences among treatments and controls were associated with AF3357 when exposed to β-caryophyllene treatments, whereby AF3357 spore concentrations were comparable to those of AF36 (Fig. 8a). Regarding spore size, we observed no major differences among treatments compared to controls for either strain (Fig. 8a). VOC treatment led to a reduction in aflatoxin content in AF3357 strain that was only significant with 1 M β-caryophyllene compared with DMSO (Fig. 8b). Although a general trend can be observed leading to a reduction in toxin content compared to both control and DMSO (Fig. 8b), the sample variation was too high to lead to statistically significant differences. All three β-caryophyllene treatments led to significant reduction in cyclopiazonic acid (CPA) and ditryptophenaline in AF3357 and AF36 compared to both control and DMSO (Fig. 8b). AF3357 also produced high concentrations of toxic indole diterpenes that typically concentrate in sclerotia, including aflatrem, aflavine and aflazole, but these levels were unaffected by VOC treatments (Supplemental Fig. 6).

## Discussion

*A. flavus,* an opportunistic pathogen that is ubiquitous in nature and soil is able to emit VOCs whose production, regulation, biological and ecological role are not fully understood. Guo et al. (2021) examined 43 fungal species and reported that VOC profiles can predict phenotypic traits, such as trophic modes/lifestyles and determined that saprotrophs have significant production of monoterpenoids (Guo et al. 2021), terpenes with a 10-carbon structure (González-Hernández et al. 2023). The monoterpenoids detected by Guo et al. (2021) did not show significant association with the *A. flavus* (opportunistic saprotrophic lifestyle) (Guo et al. 2021), isolates we examined in this study, although sclerotia grown on soil produced significantly high levels of geranyl acetone, a monoterpene ketone, and caryophyllene (Fig. 1 and 2, Supplemental Table 1). Although Guo et al. (2021) assessed VOCs from several saprotrophic fungi, they did not study *A. flavus* (Guo et al. 2021). Instead, the saprotrophic fungi examined were *Alternaria alternata, Aspergillus niger,* and *Aspergillus oryzae*, a closely related nonaflatoxigenic fungus to *A. flavus.* Among those three species, *A. alternata* (multitrophic lifestyle preference) produced sesquiterpenoids such as, β-caryophyllene and isocaryophyllene, which are similar to the ones we detected in *A. flavus* (Supplemental Table 1). The secondary metabolism of species having multitrophic lifestyle preferences is adaptable, leading to changes in VOC production linked to environmental cues (Guo et al. 2021). Therefore, it is possible that the opportunistic multitrophic lifestyle of *A. flavus* is linked to variable and distinct signatures of VOCs that can vary in relation to the substrate on which they grow.

In this study, we determined that *A. flavus* sclerotia grown in soil produced significantly higher amounts of caryophyllene and caryophyllenyl alcohol VOCs compared to conidia grown in soil and control treatments (Fig. 5 and 6; Supplemental Table 1). Caryophyllene is a terpene VOC classified as a sesquiterpenoid due to its 15-carbon structure (González-Hernández et al. 2023; Quin et al. 2014). For *A. flavus*, most VOCs were previously identified while growing on synthetic media (Moore and Lloyd 2023, 2025; Josselin et al. 2022; Josselin et al. 2021) but not while growing in soil. For example, aflatoxigenic strains of *A. flavus* grown in Adye and Mateles liquid medium produced α-gurjunene, *trans*-caryophyllene, and cadinene at 3 days post-inoculation (dpi), none of which were captured in the headspaces of non-aflatoxigenic strains (Zeringue et al. 1993). Other studies have shown that, when grown in Potato Dextrose Agar (PDA), aflatoxigenic strains of *A. flavus*, emitted terpene VOCs such as epizonarene, *trans*-caryophyllene, valencene, α-copaene, β-himachalene, γ-cadinene, γ-muurolene, δ-cadinene (Josselin et al. 2022; Josselin et al. 2021). Terpenoids are a diverse family of secondary metabolites with myriad biological roles (Quin et al. 2014; Hilgers et al. 2021). Their roles in fungi include electron transport, cell wall and membrane formation, chemical defense against predators and messengers during symbiotic relationships with other organisms (González-Hernández et al. 2023; Quin et al. 2014). Characterizing the function(s) of caryophyllene compounds would advance our understanding of their biological and ecological roles in *A. flavus.*

Sclerotia of toxigenic *A. flavus* strain AF3357 produced caryophyllene compounds (Fig. 5 and 6) while in a soil environment. Studies have shown that in corn, infestation by larvae of the African cotton leafworm (*Spodoptera littoralis*) triggers the plant’s production and accumulation of *trans*-β-caryophyllene, which attracts natural enemies of *S. littoralis* (Robert et al. 2012a; Robert et al. 2012b). Similarly, this corn-produced VOC deters western corn rootworm (*Diabrotica virgifera*) infestations in the soil environment by attracting natural enemies of *D. virgifera*, such as entomopathogenic nematodes (Robert et al. 2012b; Rasmann and Agrawal 2008; Rasmann et al. 2005; Capra et al. 2015; Kollner et al. 2008). In summary, caryophyllene is a key player in the complex chemical communication between plants and insects, helping corn plants defend themselves and manage their interactions with the environment (Kollner et al. 2008). Given the evidence of caryophyllene’s biological role in corn, it is possible that it could play similar roles in fungi, such as communication and signaling (Razo-Belmán et al. 2023). Further research will aid understanding the biological and ecological role(s) that caryophyllene and similar VOCs play in *A. flavus*. Given that AF3357 can produce and respond to β-caryophyllene, we hypothesize that there is a multitrophic interaction effect among corn-insects-fungi involving multiple VOCs. The enhanced production of caryophyllene and other compounds in fungal sclerotia may serve as a deterrent to fungivorous insects so that the overwintering structure remains intact until conditions are favorable for germination. Future studies should test the impact of *A. flavus* sclerotia, especially those that produce an abundance of caryophyllene, on insects that prey on fungal tissue.

We determined that β-caryophyllene can modulate aflatoxin production in AF3357 by significantly decreasing its abundance compared to DMSO. The inhibitory potential of β-caryophyllene on aflatoxin production has been shown through the use of essential oils extracted from *Zanthoxylum armatum,* which contained 7.53% of β-caryophyllene, used to treat stored seeds of *Platycladus orientalis* (L.) Franco (Li et al. 2021). Treatment with this oil on seeds infected with *A. flavus* reduced fungal mass, colony number and aflatoxin levels (Li et al. 2021). Other essential oil-based studies using extractions from pepper (*Piper nigrum* L.) and clove (*Eugenia caryophyllata*) that contained high levels of β-caryophyllene inhibited growth of *Fusarium oxysporum* and *Aspergillus niger* by 40–70% (Muñoz Castellanos, et al. 2020). Furthermore, pharmacological studies showed that the addition of β-caryophyllene to aflatoxin B_1_ treatments led to improved liver parameters in Wistar rats, indicating the potential for β-caryophyllene to reduce aflatoxin B_1_ induced hepatotoxicity (Silveira et al. 2021). It is possible, therefore, that production of β-caryophyllene is a self-regulation mechanism for growth and mycotoxin production in mycotoxigenic fungi like *A. flavus*. Although the biocontrol strain AF36 does not produce aflatoxin, it still produces other fungal mycotoxins such as CPA (Chang et al. 2009; Chalivendra et al. 2017), and ditryptophenaline (Barrow and Sedlock 1994; Springer et al. 1977). We determined that β-caryophyllene can significantly decrease the production of both CPA and ditryptophenaline in both AF36 and AF3357. To our knowledge, this is the first time that caryophyllene compounds have been shown to inhibit the production of mycotoxins (i.e., aflatoxin, CPA), as well as the secondary metabolite ditryptophenaline, by *A. flavus*. The implications that this discovery has for fungal ecology and clinical fields are yet to be determined. Research has shown that ditryptophenaline has antimicrobial activity (Abd El-Rahman et al. 2020; Singab et al. 2023), which could serve as an ecological advantage to *A. flavus* when competing for resources against other microorganisms, potentially making β-caryophyllene a key player in the modulation of fungal-microbe-plant interactions.

We have identified that conidia and sclerotia from AF3357 growing in soil produced caryophyllene compounds and that their production was greater in sclerotia. We observed differences in phenotypic responses to β-caryophyllene exposure between AF3357, an aflatoxin producing strain, and AF36, a non-aflatoxin producing strain (commercial biocontrol agent). Like AF3357, the AF36 strain produces conidia and sclerotia that are considered L-type (*i.e.*, large sclerotia), having diameters greater than 400 µm (Cotty 1989). Whether or not AF36 conidia and/or sclerotia produce caryophyllene, or other VOCs that overlap with AF3357 VOC profiles, while growing on soil, has yet to be assessed. Exposure of these strains to 3.5 M β-caryophyllene showed AF3357’s perception of this compound triggered a decrease in production of aerial mycelia and an increase in spore production to a point that was at par with AF36 (Fig. 7). We observed AF3357 had a faster growth rate than AF36, which we observed with every VOC treatment and control (Fig. 8a). This was an unexpected result because AF36 is considered an aggressive colonizer (Cotty 1989). However, AF36 does not necessarily need to grow faster to be more effective at colonization. Although AF3357 grew faster, AF36 produced significantly more spores (Fig. 8a). This may be one of the reasons AF36 is an effective biocontrol strain in the field, its enhanced spore production compared to AF3357 facilitates better dispersal than its slower growing mycelia can travel, and more abundant inoculum (*i.e.*, asexual propagules) ensures greater chances of survival (Horn et al. 2016; Erental et al. 2008).

Concomitantly, the phenotypic responses from AF3357 and AF36 that result in greater production of conidia as asexual propagules for better dispersion and survival could also facilitate an abundance of conidia as male gametes for sexual reproduction. Not only is production of sclerotia by *A. flavus* key in overwintering during unfavorable environmental conditions, but they also serve as female (receptor) gametes for sexual outcrossing while conidia serve as male (donor) gametes (Horn et al. 2016). Thus, the hermaphroditic nature of *A. flavus* strains that produce both conidia and sclerotia is a key evolutionary tool for ensuring its adaptability and survival. Based on the impacts of these compounds on conidium production, β-caryophyllene and caryophyllene oxide, being produced at greater concentrations in sclerotia, could serve as semiochemicals (*i.e.*, pheromones) to (1) stimulate increased production of conidia (donor gametes) in sexually compatible neighbors, and/or (2) help germinating conidia to find sclerotia (receptor gametes) in the soil environment to initiate sexual outcrossing. Further research is necessary to confirm or refute this possibility. AF36 sclerotia are reportedly sterile (Horn et al. 2016), but it has successfully contributed to the production of progeny (Horn et al. 2016; Erental et al. 2008; Willetts and Bullock 1992). AF3357 sclerotia produced an abundance of caryophyllene compounds, and these compounds stimulated vegetative growth and sporulation for this strain. Therefore, it is possible that the ‘awakening’ of a sclerotium from its dormant state also involves a chemical VOC signal, like caryophyllene, produced by itself and/or neighboring sclerotia. Additional research is required to address this hypothesis.

In summary, this VOC study revealed that sclerotia of AF3357 produced significantly higher levels of caryophyllene-related compounds compared to conidia and controls in soil (Fig. 1, 2, 3, 4, 5 and 6 and supplemental Table 1), also that β-caryophyllene modulates conidia growth (Fig. 7) and suppresses aflatoxin, CPA and ditryptophenaline production (Fig. 8b). Our results offer additional VOCs that could be used to develop methods to prevent mycotoxin contamination by detecting the presence of *A. flavus* during sclerotia and conidia developmental stages in the soil. Our VOC profiles can be used for training volatile biomarker technology to detect compounds such as monoterpenoids released by saprotrophs (Guo et al. 2021). This kind of technology could serve as the basis to detect the presence of *A. flavus*-sclerotia VOCs prior to planting and/or conidia prior to harvesting (infection-related VOCs). Here we showed for the first time VOCs from *A. flavus* conidia and sclerotia while growing in soil environments and detected sclerotia-specific VOCs that could aid further research on fungal detection biomarkers. Furthermore, we showed that β-caryophyllene and caryophyllene oxide play a role in modulating hyphal and spore production in AF3357 while not showing an effect on the biocontrol non-toxin producing fungi AF36. Also, β-caryophyllene can modulate aflatoxin production. The consequences that VOC-modulated developmental changes and mycotoxin production have on the ecological impacts of toxin-producing and non-toxin producing fungi can be key for the corn-fungal interactions. We hypothesize that the biological role of β-caryophyllene in *A. flavus* might be involved in development, reproduction and defenses. Further research in this ecological role would be helpful in addressing the serious concern of mycotoxin contamination in corn and the potential involvement in multitrophic interactions in the field.

## Supplementary Information

Below is the link to the electronic supplementary material.

Sup. Figure 1 (PNG 2.36 MB)

High Resolution Image (TIF 3117 KB)

Sup. Figure 2 (PNG 273 KB)

High Resolution Image (TIF 537 KB)

Sup. Figure 3 (PNG 294 MB)

High Resolution Image (TIF 568 KB)

Sup. Figure 4 (PNG 461 MB)

High Resolution Image (TIF 724 KB)

Sup. Figure 5 (PNG 278 MB)

High Resolution Image (TIF 390 KB)

Supplementary File 6 (JPG 360 KB)

Supplementary File 7 (XLSX 21.1 KB)

## Data Availability

No datasets were generated or analysed during the current study.
