# Supplementary figures and images for: Volatilized Metabolites Produced by Soilborne Aspergillus flavus Regulate Fungal Conidiation, and Production of Secondary Metabolites
Source: J Chem Ecol. 2026 Jan 22;52(1):12. doi: 10.1007/s10886-025-01657-4 (PMC12827406; doi:10.1007/s10886-025-01657-4)

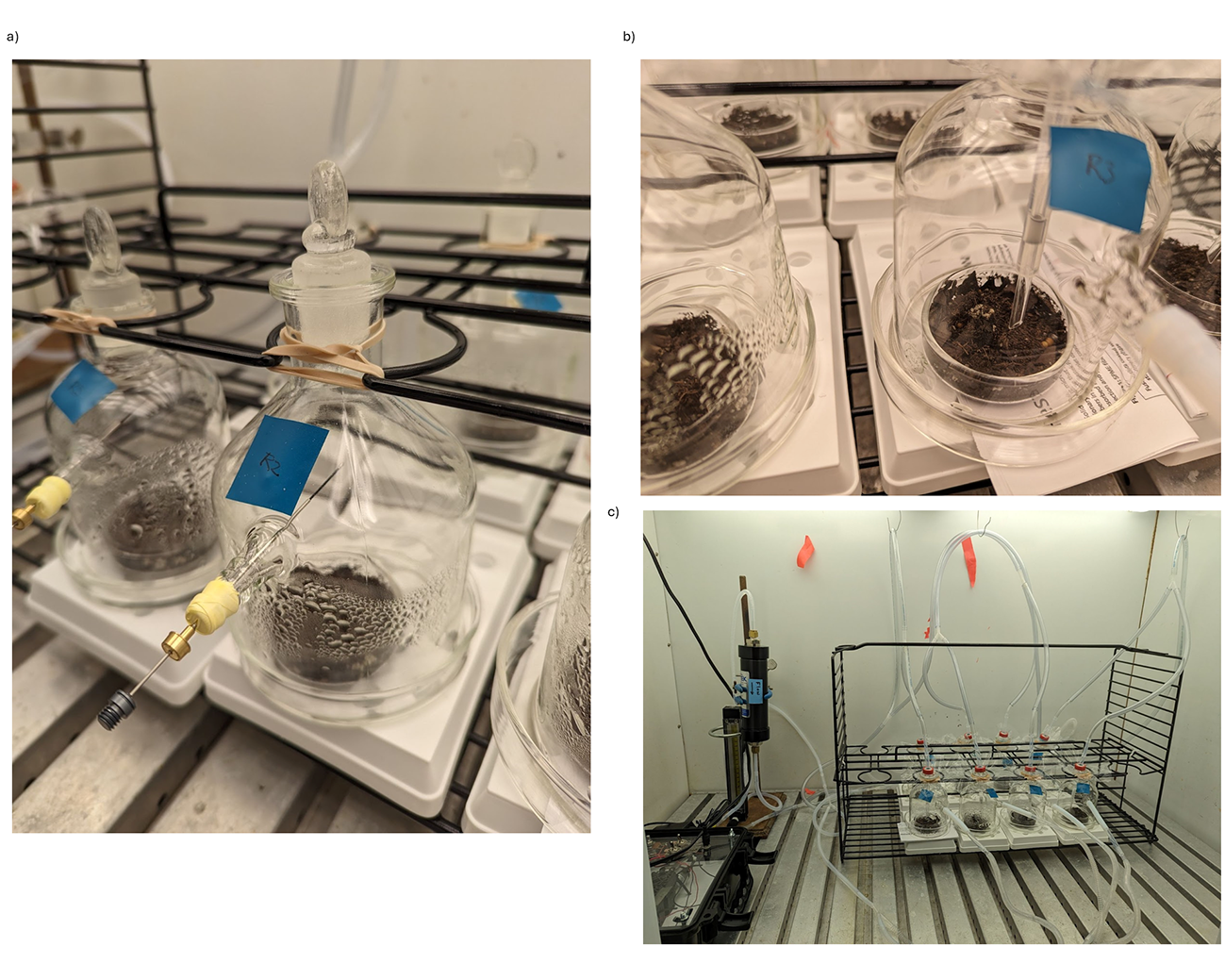

Supplement: Supplementary file 1 — (PNG 2.36 MB) [file 10886_2025_1657_Fig9_ESM.png]

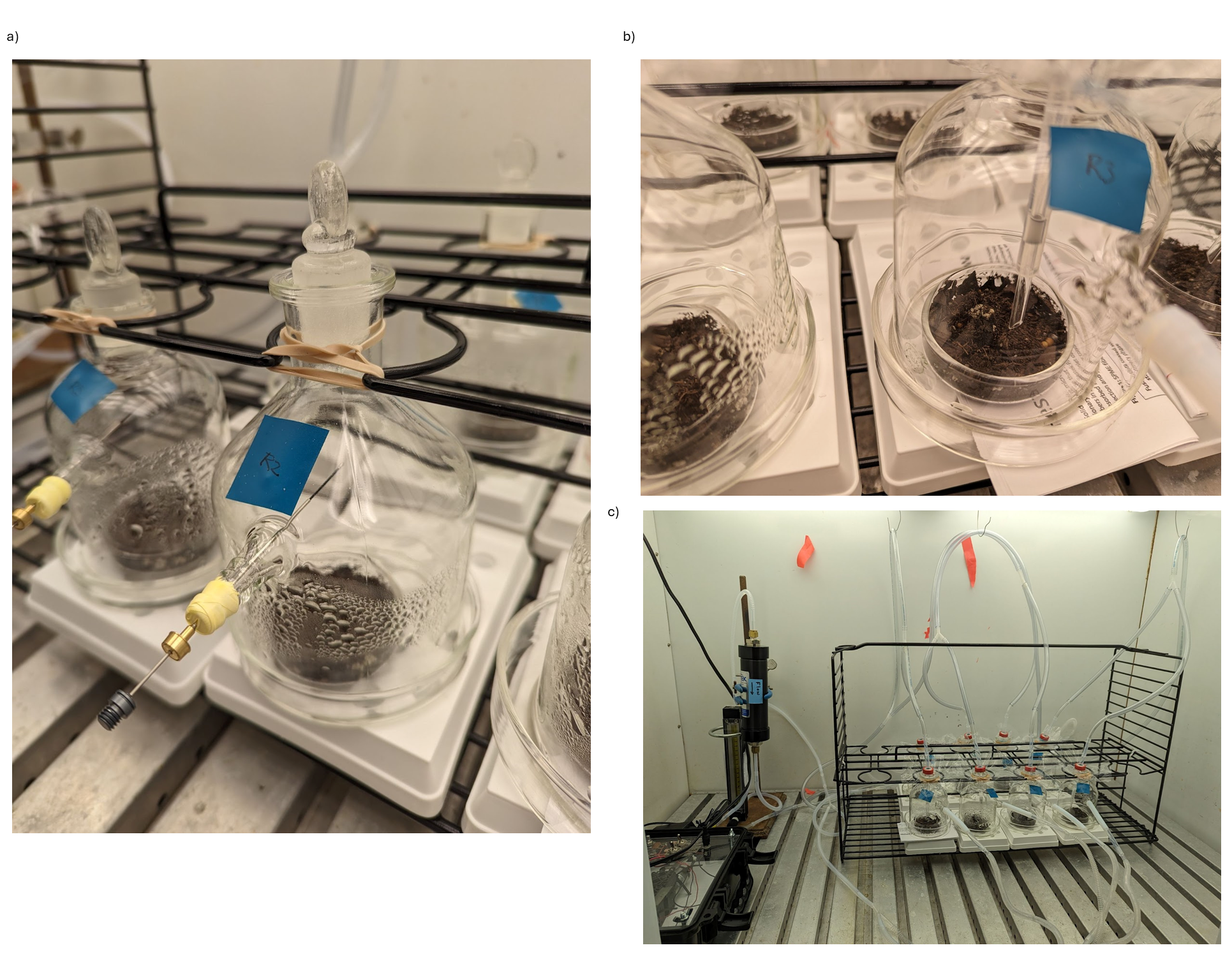

Supplement: Supplementary file 2 — High Resolution Image (TIF 3117 KB) [file 10886_2025_1657_MOESM1_ESM.tif]

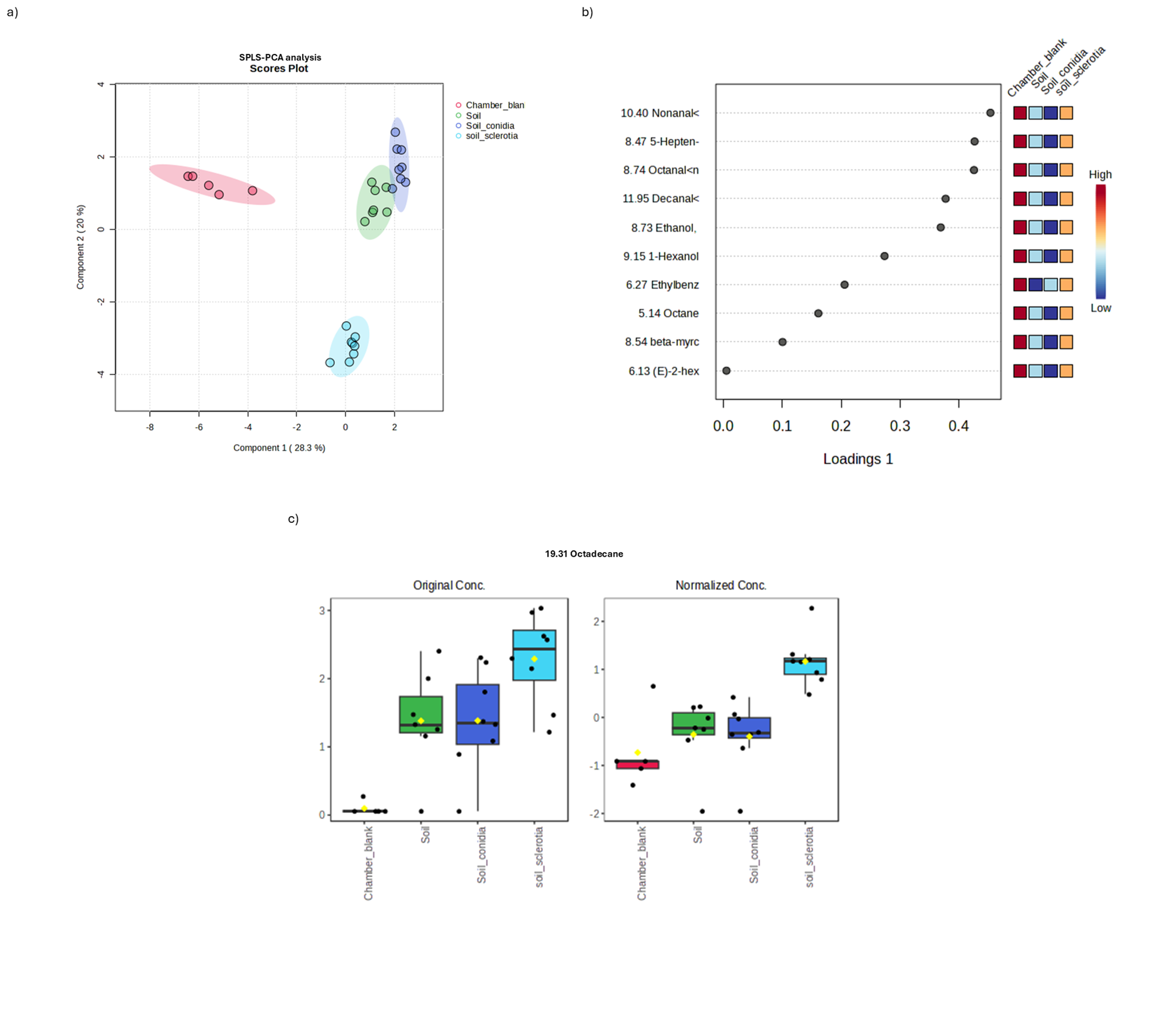

Supplement: Supplementary file 3 — (PNG 273 KB) [file 10886_2025_1657_Fig10_ESM.png]

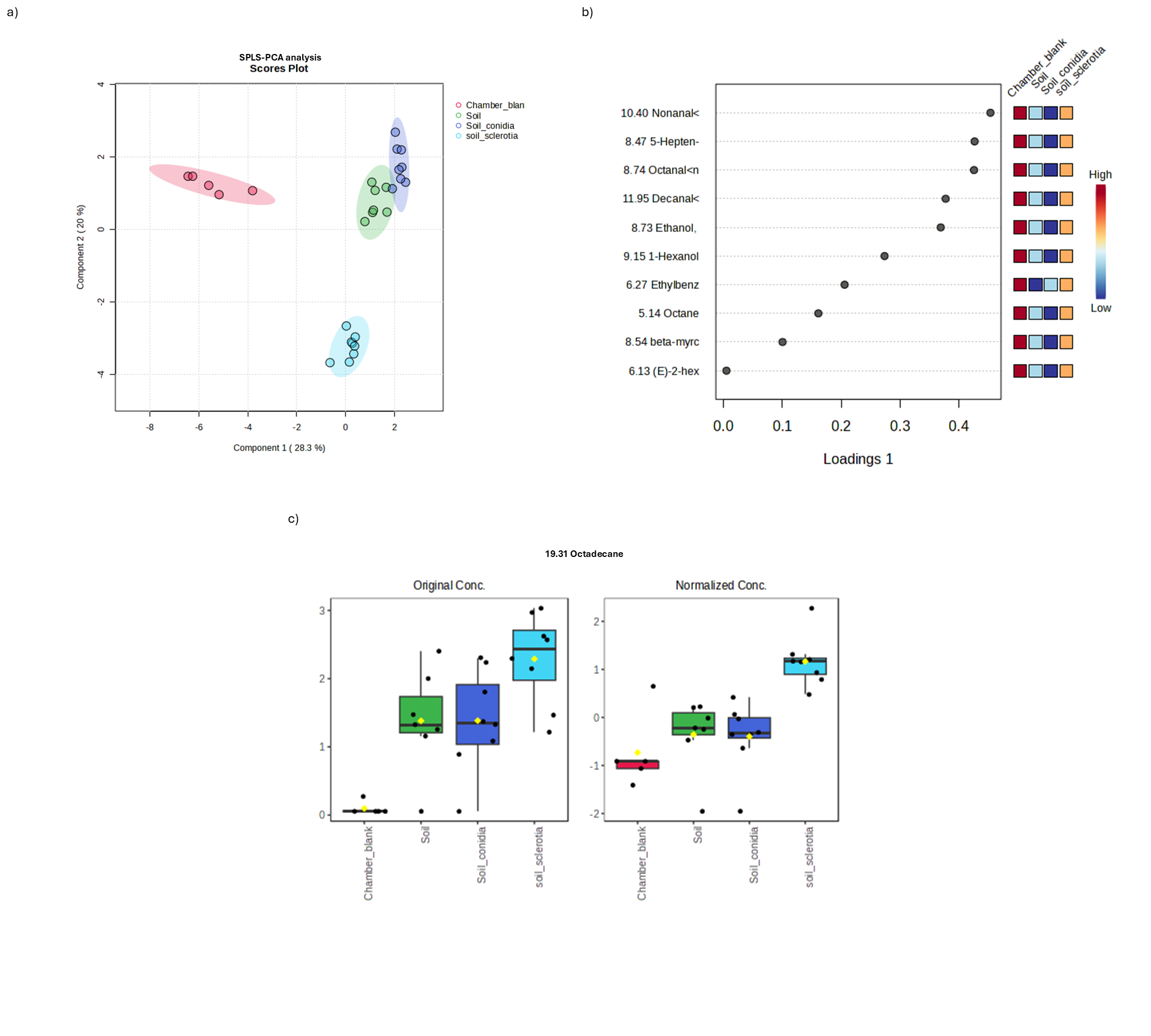

Supplement: Supplementary file 4 — High Resolution Image (TIF 537 KB) [file 10886_2025_1657_MOESM2_ESM.tif]

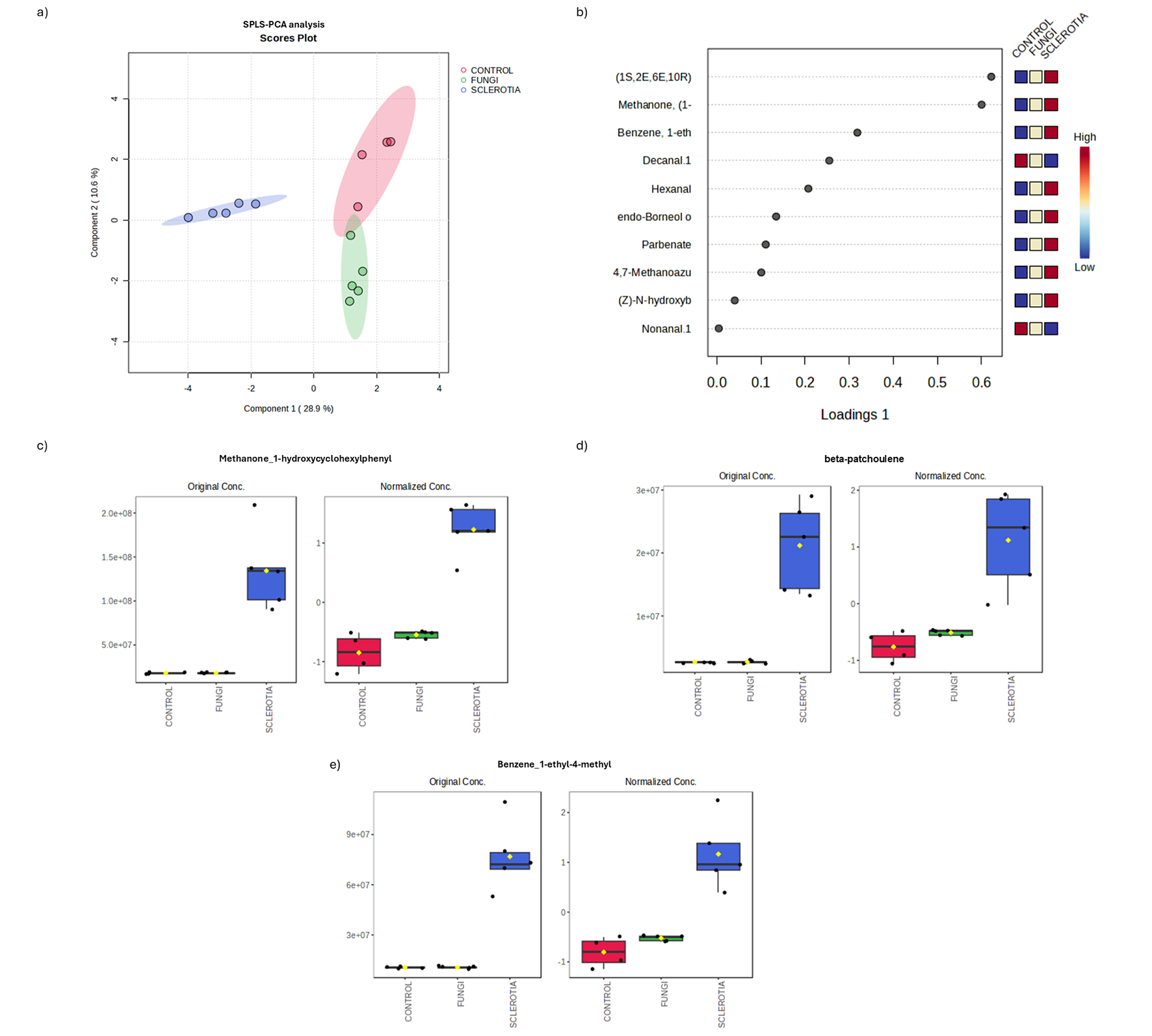

Supplement: Supplementary file 5 — (PNG 294 MB) [file 10886_2025_1657_Fig11_ESM.png]

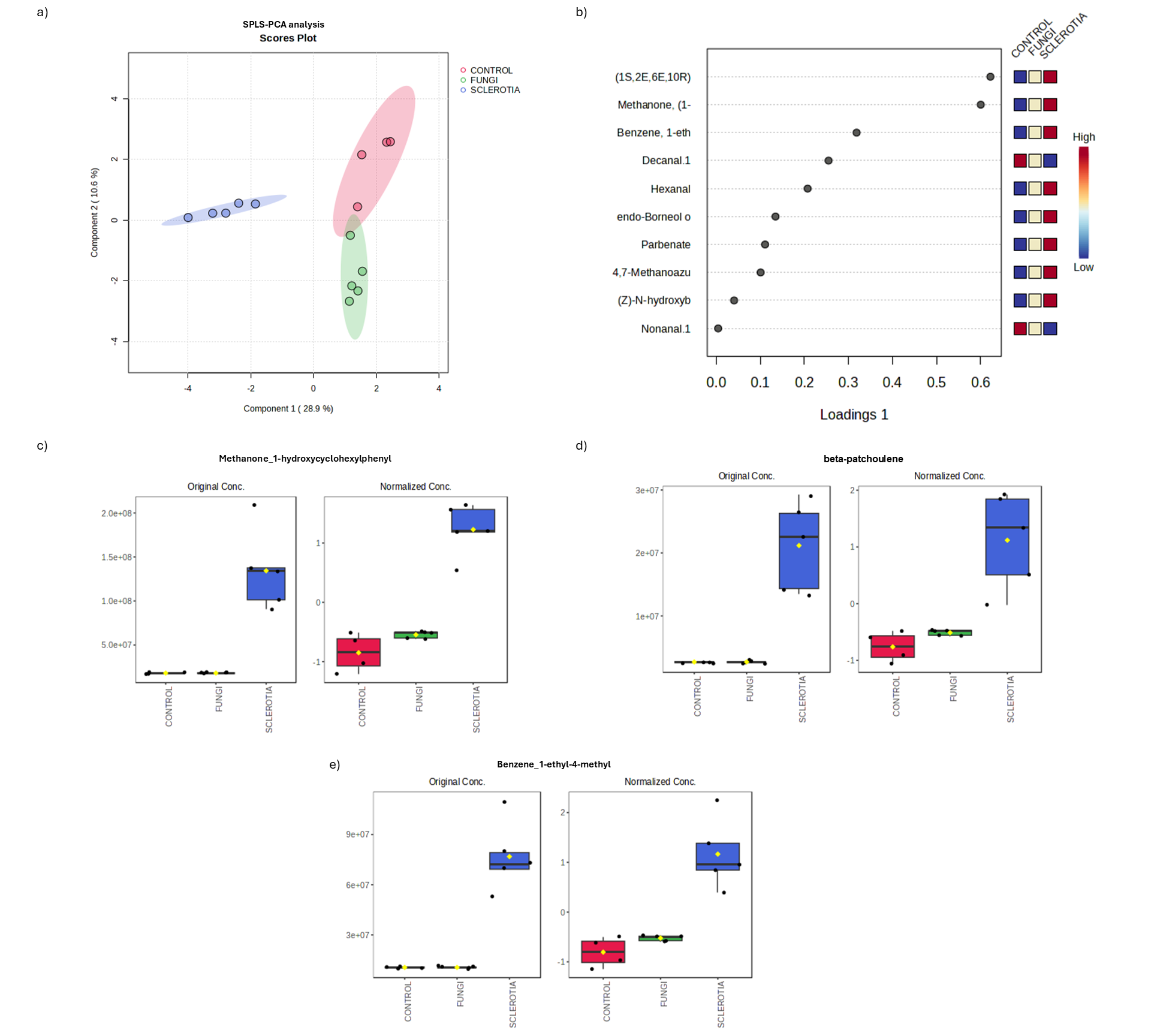

Supplement: Supplementary file 6 — High Resolution Image (TIF 568 KB) [file 10886_2025_1657_MOESM3_ESM.tif]

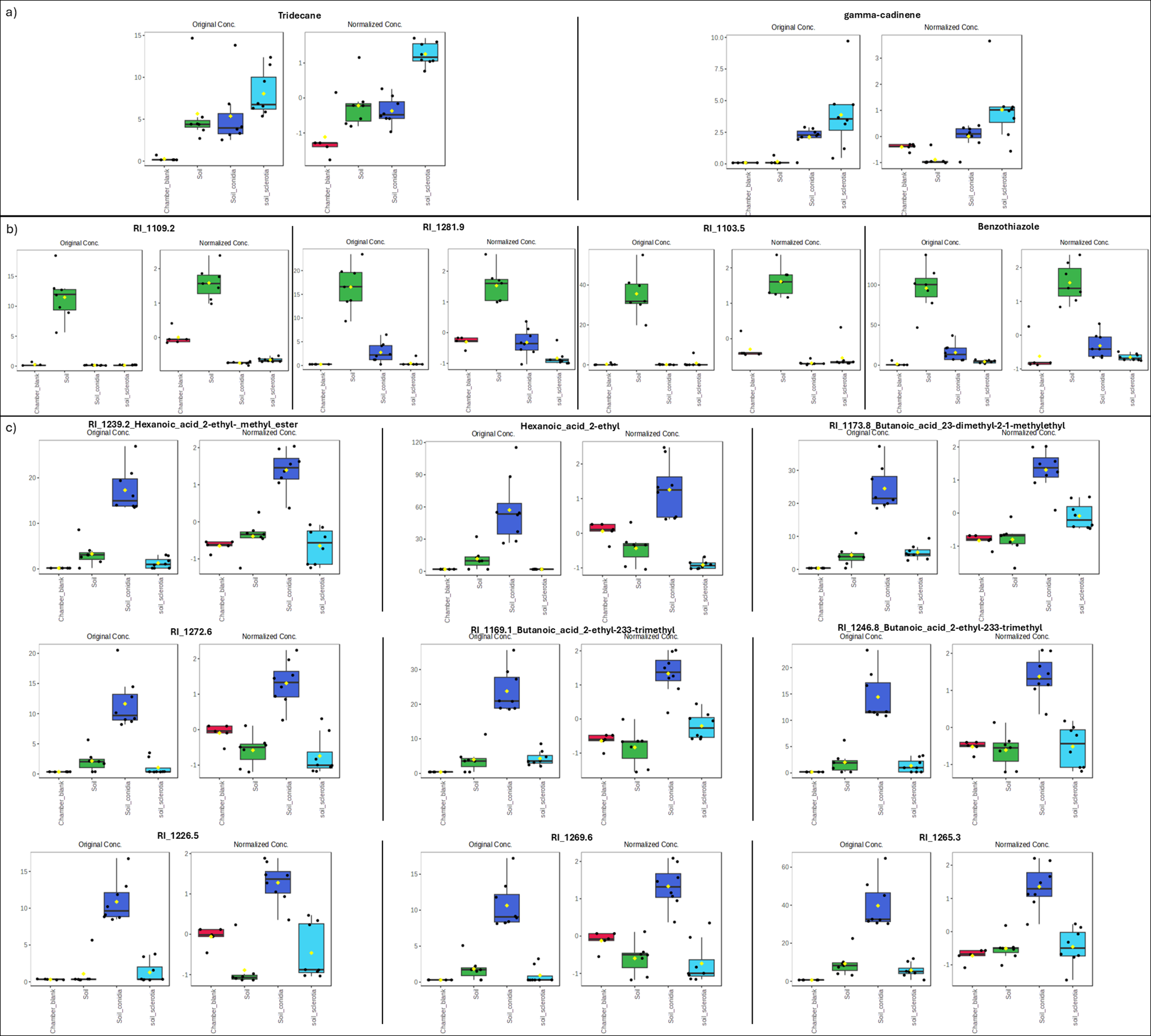

Supplement: Supplementary file 7 — (PNG 461 MB) [file 10886_2025_1657_Fig12_ESM.png]

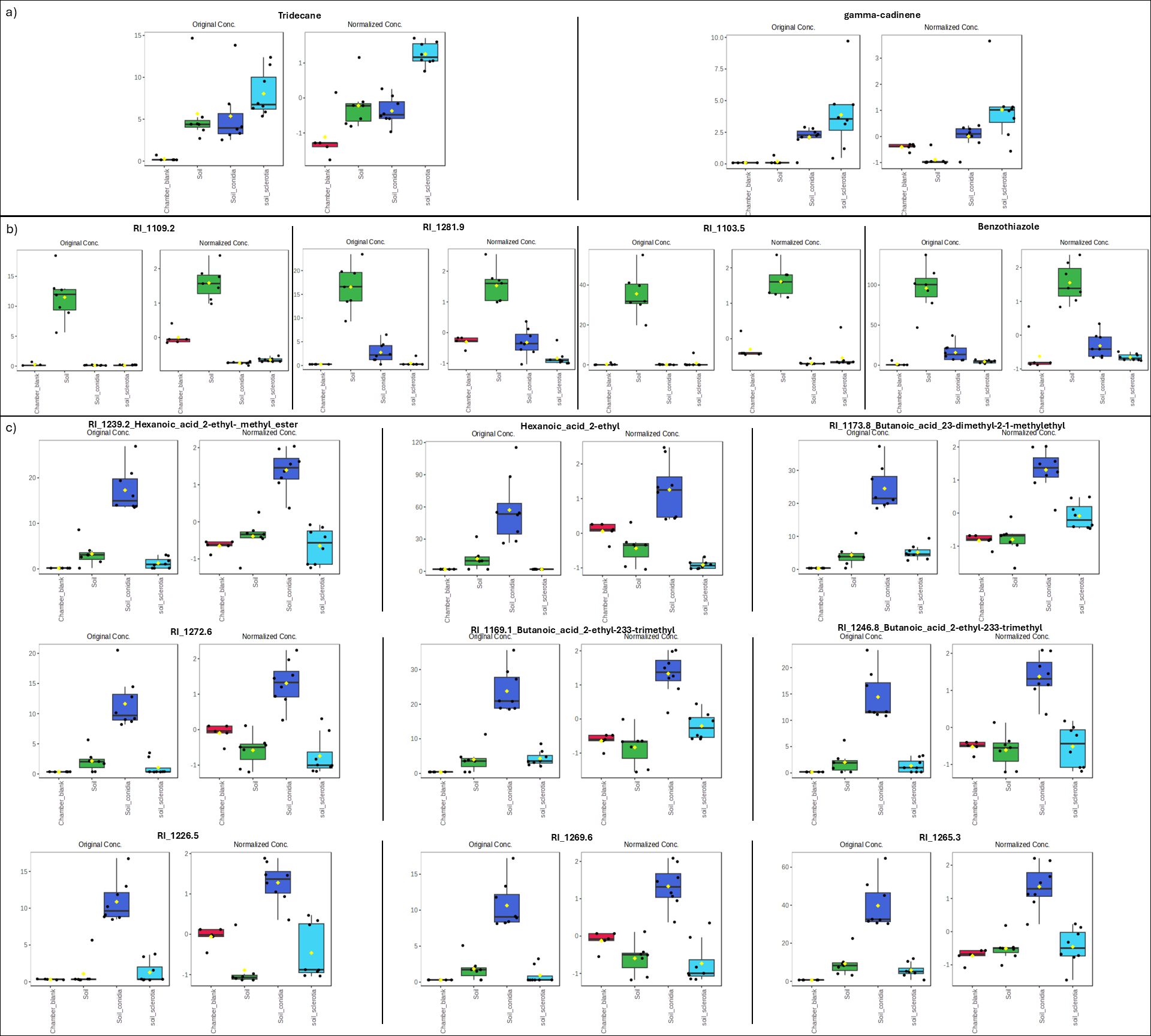

Supplement: Supplementary file 8 — High Resolution Image (TIF 724 KB) [file 10886_2025_1657_MOESM4_ESM.tif]

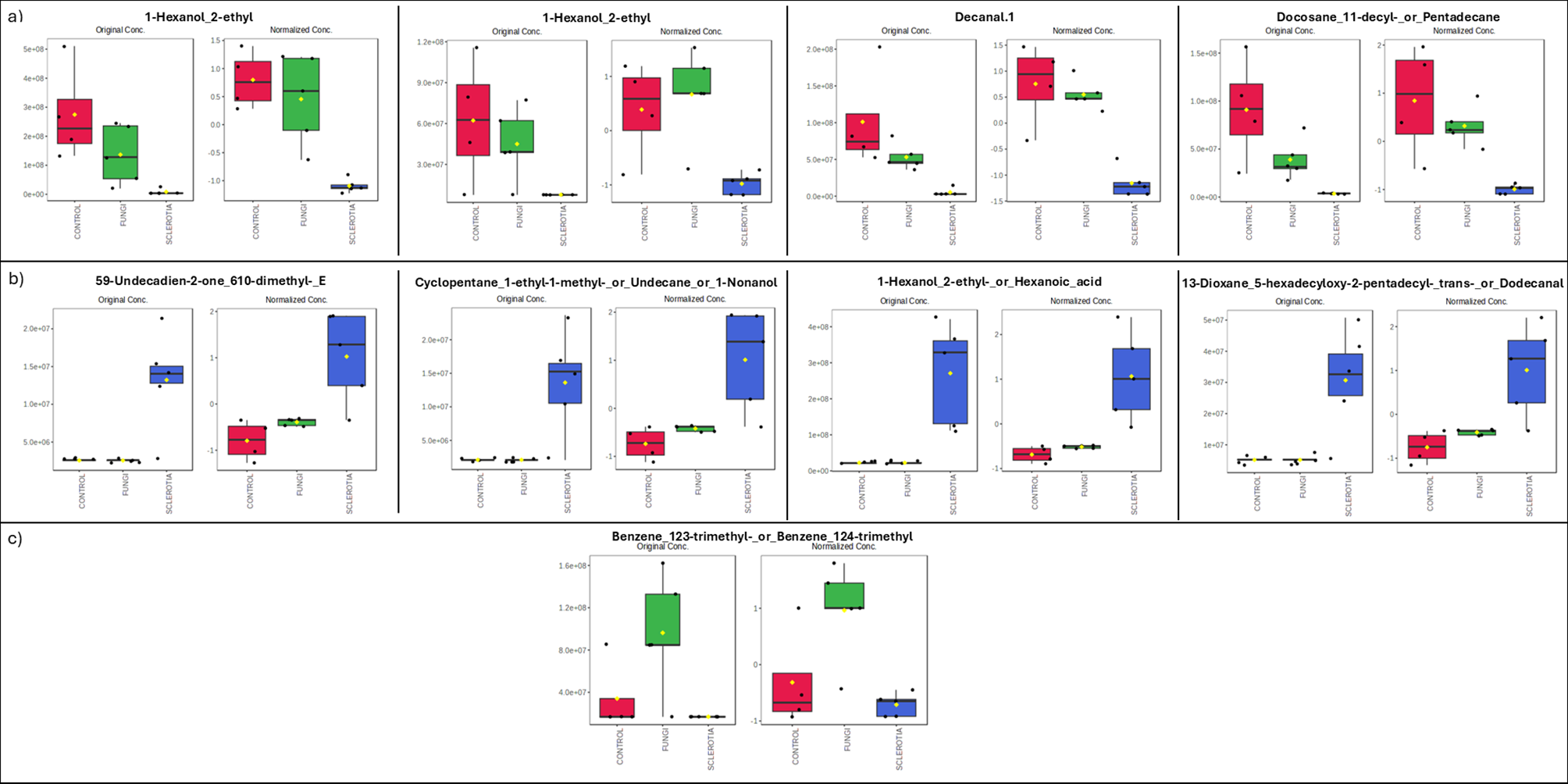

Supplement: Supplementary file 9 — (PNG 278 MB) [file 10886_2025_1657_Fig13_ESM.png]

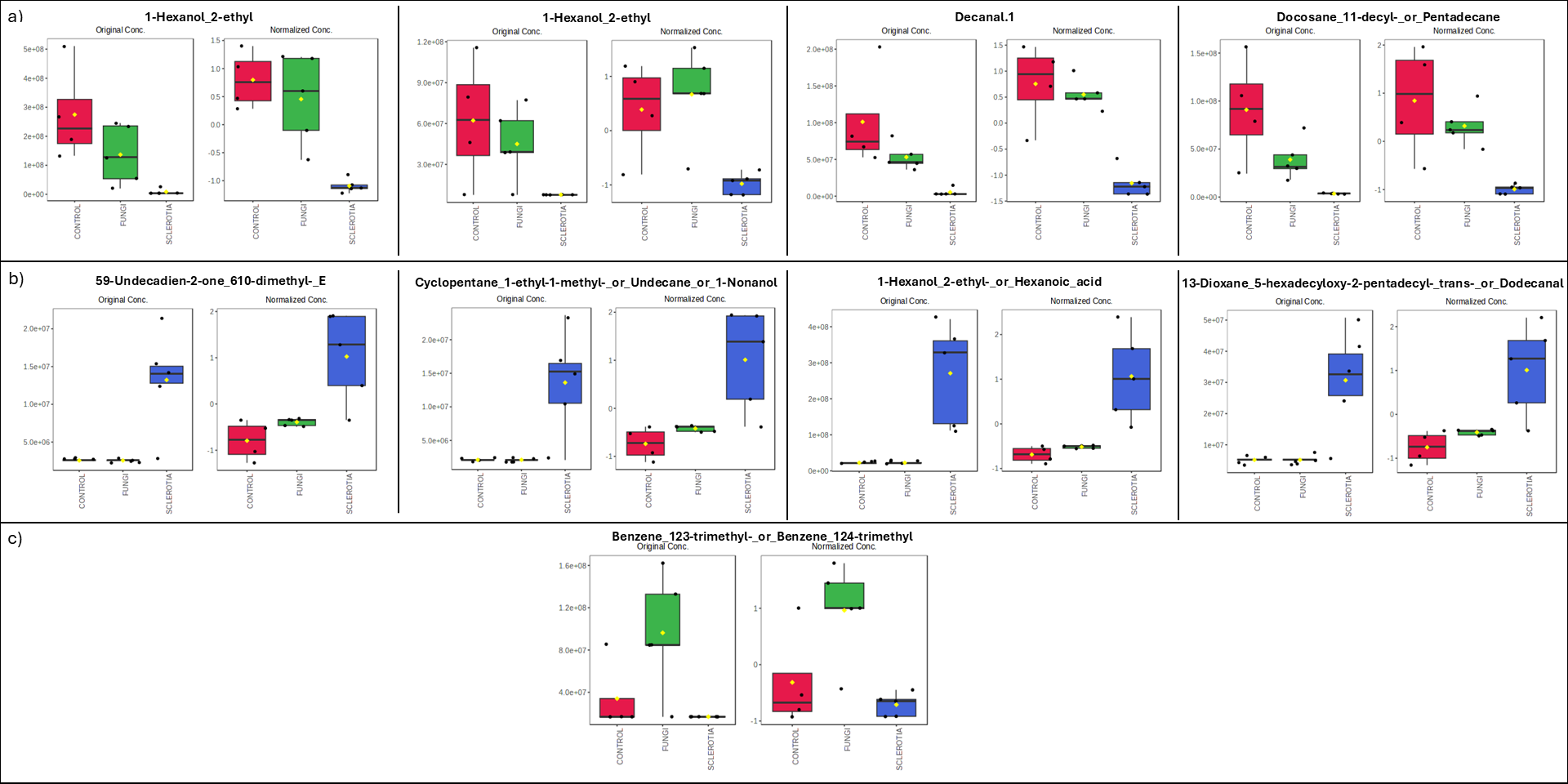

Supplement: Supplementary file 10 — High Resolution Image (TIF 390 KB) [file 10886_2025_1657_MOESM5_ESM.tif]

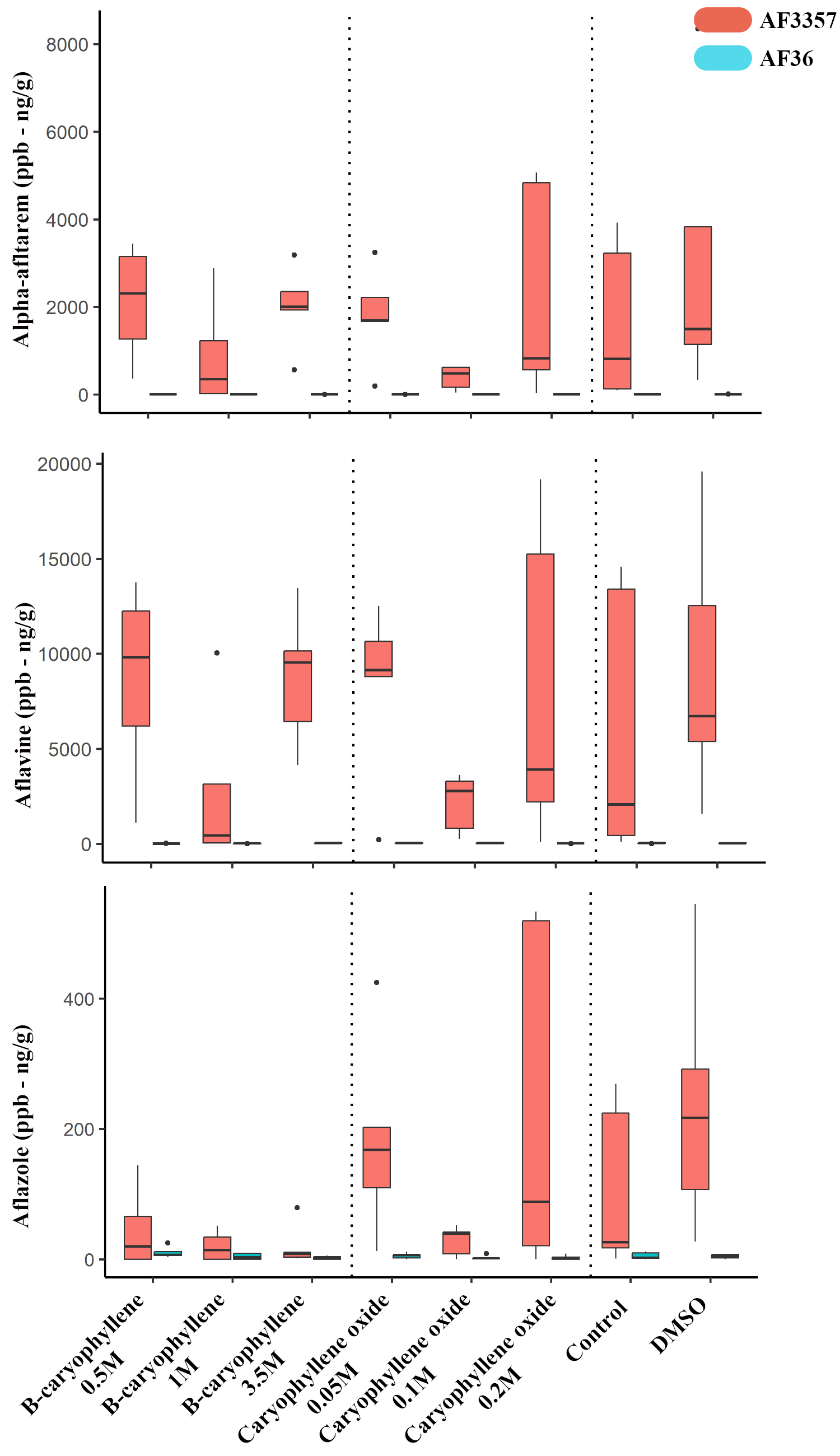

Supplement: Supplementary file 11 — Supplementary File 6 (JPG 360 KB) [file 10886_2025_1657_MOESM6_ESM.jpg]
